# Towards precision dosing of vancomycin in patients with allogeneic hematopoietic stem cell transplantation: a comparison of published population pharmacokinetic models

**DOI:** 10.1128/aac.00257-25

**Published:** 2025-08-14

**Authors:** Eva-Maria A. Wansing, Emily Behrens, Nicolaus M. Kröger, Claudia Langebrake, Sebastian G. Wicha

**Affiliations:** 1 Hospital Pharmacy, University Medical Center Hamburg-Eppendorf 37734, Hamburg, Germany; 2 Department of Clinical Pharmacy, Institute of Pharmacy, University of Hamburg 14915, Hamburg, Germany; 3 Department of Stem Cell Transplantation, University Medical Center Hamburg-Eppendorf 37734, Hamburg, Germany; Providence Portland Medical Center, Portland, Oregon, USA

**Keywords:** vancomycin, pharmacokinetics, stem cell transplantation, model-informed precision dosing, therapeutic drug monitoring, Bayesian, population pharmacokinetics

## Abstract

Patients undergoing allogeneic hematopoietic stem cell transplantation (allo-HSCT) often receive vancomycin as antibiotic treatment using therapeutic drug monitoring (TDM). TDM can be performed using a Bayesian approach, where concentration measurements are supported by a previously developed pharmacokinetic model based on a population as similar as possible to the treated patient. As a model developed in a similar population is not always available, it must be ensured that the predictive performance is not compromised. We retrospectively collected data from 121 adult allo-HSCT patients who received vancomycin treatment including trough concentration TDM between January and December 2021. Predictive performance of 21 published pharmacokinetic models was assessed by comparing the third observed vancomycin concentration per patient with the corresponding model prediction. Prediction was based on covariates alone (*a priori*) or covariates and TDM measurements (Bayesian). Predictive performance was quantified by a median prediction error (MPE) for accuracy and median absolute prediction error (MAPE) for precision. MPE ranged between −199.6% and 93.3% (*a priori*) and between −50.6% and 19.1% (Bayesian), while MAPE ranged between 31.6% and 199.6% (*a priori*) and between 19.8% and 53.5% (Bayesian). The Okada et al. model was one of the most accurate and precise models in the *a priori* (MPE: −4%; MAPE: 31.6%) and Bayesian scenario (MPE: −6.9%; MAPE: 19.8%). The model published by Okada et al. was developed in allo-HSCT patients. That may explain the high predictive performance, especially in the *a priori* scenario. We recommend the Okada et al. model for future TDM in allo-HSCT patients.

## INTRODUCTION

Infection is the second most common cause of death after allogeneic hematopoietic stem cell transplantation (allo-HSCT), with almost 60% of cases being of unknown etiology (1). Neutropenic fever is often the only symptom, and immediate empiric antibiotic treatment is highly recommended (2–4). If identified, the most common types of infection after hematopoietic stem cell transplantation are pneumonia, gastrointestinal infections, and bloodstream infections. The latter is caused by Gram-positive bacteria in more than half of the cases (5–7). Therefore, vancomycin is commonly used as part of empirical antibiotic treatment but must be carefully dosed due to its narrow therapeutic range. Therapeutic drug monitoring (TDM) is, therefore, used and especially important for patient populations with altered pharmacokinetics (PK) (8, 9). TDM of vancomycin significantly improves patient outcome and reduces adverse drug effects, such as nephrotoxicity (10, 11).

In routine clinical care, different approaches are used to perform TDM. Trough concentration TDM is widely used because there is no need for special software, and only one sample per dosing interval is required. However, one of the limitations of this method is that it can only be applied once steady state has been reached. Furthermore, recent publications indicate that the 24-hour area under the curve (AUC_24_) above the minimal inhibitory concentration (MIC) target of >400 and AUC_24_ <600 is superior to the previously used trough concentration target (12–14). AUC-based dosing through a dosing software was associated with fewer samples per patient, shorter treatment duration, and reduced nephrotoxicity (14). Additionally, reduced rates of nephrotoxicity with AUC-based dosing strategies rather than trough-based monitoring were confirmed in recent systematic meta-analyses (15, 16).

TDM with an AUC_24_/MIC target >400 and AUC_24_ <600 can be applied using different methods. Either the AUC is calculated according to first-order PK equations using a peak and a trough concentration, or a Bayesian approach is used. For the latter, only a single blood sample is required as the estimation of the PK profile is supported by a previously developed PK model based on a population which closely resembles the treated patient. This approach is commonly referred to as model-informed precision dosing (MIPD). For MIPD in routine clinical care, the models are integrated into specialized TDM software such as TDMx (17), a validated, web-based MIPD software that allows clinicians to generate dosing suggestions based on prior information and patient-specific parameters.

Population PK parameters of vancomycin are known to vary among different patient populations, e.g., in patients with neutropenia or cancer. However, the findings regarding PK differences between neutropenic or cancer patients and the general population are variable across studies. Many cases report an approximately 30% increase in vancomycin clearance in patients with neutropenia, necessitating higher vancomycin doses to achieve an adequate AUC (18–21). The volume of distribution was significantly increased in some studies (22, 23), but no significant difference was found in others (20, 21). Overall, the available data is heterogeneous, and the underlying mechanisms of these PK changes are yet to be elucidated.

A specific model developed in a patient population that matches the patient to be treated is not always available, but it is imperative to use an appropriate model to ensure that the model’s predictive performance is not compromised (24, 25).

To date, several population models have been developed to describe the PK of vancomycin in different patient populations, including cancer (22, 26, 27), neutropenic (20), pediatric (28), geriatric (29), critically ill (30–33), and burn (34) patients. In a pooled analysis, Colin et al. developed a PK model based on data from 14 different patient populations (35). This proved to be one of the most appropriate models in a model comparison for patients receiving continuous vancomycin infusions (24, 35). Data on vancomycin PK in allo-HSCT patients are limited (36, 37), and there is only one population PK model developed specifically for this patient group (36). Yet, the applicability of this model in all allo-HSCT patients comes with limitations as it was developed in a relatively small group of patients, excluding individuals with acute kidney injury.

Therefore, the aim of this study is to compare published population PK models for vancomycin with regard to their suitability for patients undergoing allo-HSCT.

## MATERIALS AND METHODS

### Study design and population

We retrospectively collected data from adult patients with allo-HSCT who received vancomycin therapy during an inpatient stay at the Department of Stem Cell Transplantation at the University Medical Center Hamburg-Eppendorf (UKE) between January and December 2021. For this retrospective observational study, an ethics waiver was granted by the Ethics Committee of the Hamburg Medical Association (2022-300185-WF). Vancomycin was administered according to institutional guidelines: the loading dose was administered according to individual body weight at approximately 20 mg/kg total body weight (TBW), taking into account individual clinical parameters, while the maintenance dose was determined according to daily trough concentration TDM. Inclusion criteria were a minimum age of 18 years, an inpatient stay of at least 14 days, vancomycin treatment including documented trough concentrations from routine TDM, and at least one documented vancomycin concentration within 10 days before or after a documented serum cystatin C and serum creatinine concentration. Exclusion criteria were age below 18 years and dialysis during vancomycin treatment. Three vancomycin concentrations per patient were used for the analysis. Preference was given to trough concentrations, where cystatin C and serum creatinine concentrations were available on the same day. Data from patients with less than three vancomycin concentrations were nevertheless considered to inform the models. In addition, the following data were collected from the electronic health record: time and dose of drug administration, vancomycin concentrations, age, sex, weight, height, serum creatinine, serum cystatin C, serum albumin, C-reactive protein, and absolute leukocyte count. If the leukocyte count was less than 1 × 10^9^/L, the patient was defined as neutropenic, which was implemented as a categorical variable.

Vancomycin serum concentrations were quantified using a particle-enhanced turbidimetric inhibition immunoassay (Atellica CH Analyzer, Siemens Healthineers, Forchheim, Germany), with a detection range from 3 to 50 mg/L.

### Model screening

Published population PK models for vancomycin were screened in a systematic literature search in the online database PubMed using the search terms "vancomycin" AND "pharmacokinetics" AND "model" NOT "infants" NOT "children". A total of 1,005 results were screened with the aim of identifying population PK models developed in patient populations whose PK might be transferable to the enrolled patient population. Models developed predominantly in non-adult patients, such as neonates, children, or infants, were excluded. The remaining models were screened for population size, population type, and the PK modeling approach. Models developed with a population size of fewer than 50 patients, those developed using a nonparametric approach, or those missing mandatory information needed to recode the model were excluded. The following patient groups were considered presumably inappropriate: surgical, geriatric, burn, obese, trauma, septic/septic shock, kidney transplant patients, or patients undergoing continuous renal replacement therapy. Further information regarding the reasons for exclusion can be found in Table S1. Data processing and evaluation were performed in R (version 4.3.2) (38). PK models were recoded and processed in the nonlinear mixed effects modeling software NONMEM (Version 7.5; Icon Development Solutions, Ellicot City, MD, USA).

### Model evaluation

To assess model performance, vancomycin concentrations were predicted using the recoded population models and compared with observed concentrations from previous TDM measurements. A dosing occasion was defined as a dosing interval with observed vancomycin concentrations. Dosing occasions were not necessarily consecutive.

The third dosing occasion per patient was used as the reference occasion for comparison. The scenarios under consideration included the following: *a priori* prediction of the TDM measurement in the reference dosing occasion was performed using only patient covariates and without prior observed vancomycin concentrations.

Three Bayesian forecasting scenarios were performed using patient covariates and observed concentration data from the first, second, or both prior dosing occasions. General model fit was assessed by including all three TDM measurements to determine the best possible predictive performance of each model. The metrics used to quantify this predictive performance were the median prediction error (MPE) as a measure of accuracy and the median absolute prediction error (MAPE) as a measure of precision, using the following equations:


$$MPE=median{\left(\frac{{predicted}_{i}-{observed}_{i}}{\left(\left({predicted}_{i}+{observed}_{i}\right)/\;2\right)}\right)}^{*}100$$

$$MAPE=median|{\left(\frac{{predicted}_{i}-{observed}_{i}}{\left(\left({predicted}_{i}+{observed}_{i}\right)/\;2\right)}\right)}^{*}100|$$

There is no widely accepted threshold for MPE and MAPE, but the closer the values of the model prediction and the reference “true” value (third observed vancomycin concentration per patient), the better the model performance. Accordingly, the closer the MPE and MAPE are to zero, the better the model performance.

For additional model evaluation, prediction-corrected visual predictive checks (pcVPC) were performed and evaluated for model misspecifications.

The models with the best predictive performance in allo-HSCT patients were considered for integration into the validated, web-based MIPD software TDMx (17) for future use in routine clinical care.

Due to the wide distribution of BMI in the study population, especially in the high BMI ranges, an additional sub-analysis was performed. For this sub-analysis, the model evaluation described above was stratified by BMI, with separate analysis for patients with BMI less than 30 and those with BMI greater than or equal to 30.

Anonymized data can be provided upon reasonable request.

## RESULTS

### Study population

A total of 345 vancomycin concentrations collected from 121 adult patients with hematopoietic stem cell transplantation were analyzed. Three trough concentrations were available for 106 patients (88%), two for 12 patients (10%), and one for three patients (2%). Accordingly, data from 106 patients were used for model evaluation in the five scenarios mentioned above, and the data from 121 patients were used for pcVPCs. All patients received vancomycin as an intermittent infusion. Demographic and clinical data describing the patient population are shown in Table 1.

**TABLE 1:** Demographic and clinical data of the patient population (*n* = 121)

Characteristics	Total count (%) or median (range)
Male	73 (60)
Age (years)	58 (18–85)
Height (m)	1.76 (1.57–1.98)
Weight (kg)	76.9 (52.0–133.3)
BMI (kg/m^2^)	25.5 (18.2–47.0)
Serum creatinine (mg/dL)	0.76 (0.36–1.87)
Creatinine clearance^*a*^ (mL/min/1.73 m^2^)	103 (36–253)
Serum cystatin C (mg/L)	1.27 (0.68–2.92)
Loading dose (mg/kg)	19.2 (7.8–27.2)
Hematological disease^*b*^	
ALL	12 (9.9)
AML	37 (30.6)
Lymphoma	11 (9.1)
MDS	24 (19.8)
Myelofibrosis	19 (15.7)
Other	18 (14.9)

^*a*^ Creatinine clearance calculated using the Cockcroft and Gault formula.

^*b*^ Acute lymphocytic leukemia (ALL), acute myelocytic leukemia (AML), myelodysplastic syndrome (MDS).

### Population pharmacokinetic models

A total of 21 population PK models were included in the model evaluation (20, 22, 26, 27, 30–33, 35, 36, 39–49). The models differed in the underlying population size and type. Eleven one-compartment (1-CMT) and 10 two-compartment (2-CMT) models were tested. A measure of renal function was included as a covariate on clearance in all models. In most cases (*n* = 15), this was the creatinine clearance calculated using the Cockcroft and Gault (CG) formula (50). However, serum creatinine, cystatin C, or other formulae based on these markers were also used. Other covariates on clearance were TBW, sex, age, neutropenia, mechanical ventilation state, dialysis status, postmenstrual age, hematological disease, and central volume of distribution. For most models, a measure of weight was included as a covariate on either central or peripheral volume of distribution (*n* = 15). Others included age, sex, fat-free mass (FFM), heel prick sampling, dialysis status, and serum creatinine. Two models also included covariates for intercompartmental clearance. These were fat-free mass, heel prick sampling, and peripheral volume of distribution. Most of the models were developed for intermittent vancomycin administration, while four were explicitly developed for patients receiving continuous infusion. Characteristics describing the 21 models can be found in Table S2.

### Model evaluation

**Fig 1:**
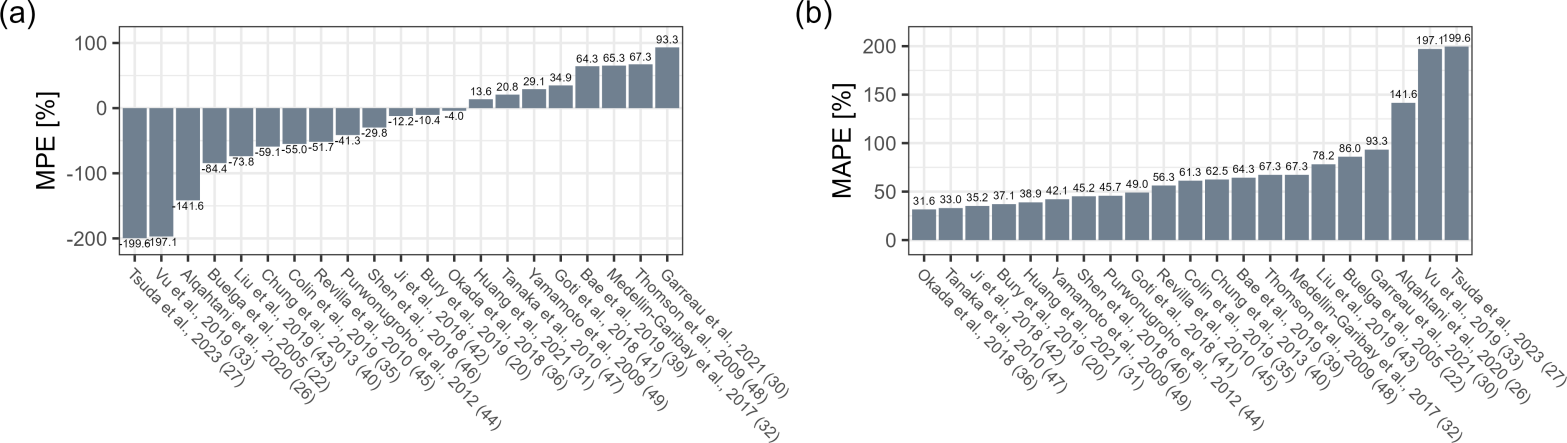
(**a**) Median prediction error (MPE [%]) and (**b**) median absolute prediction error (MAPE [%]), *a priori* scenario (20, 22, 26, 27, 30–33, 35, 36, 39–49).

**Fig 2:**
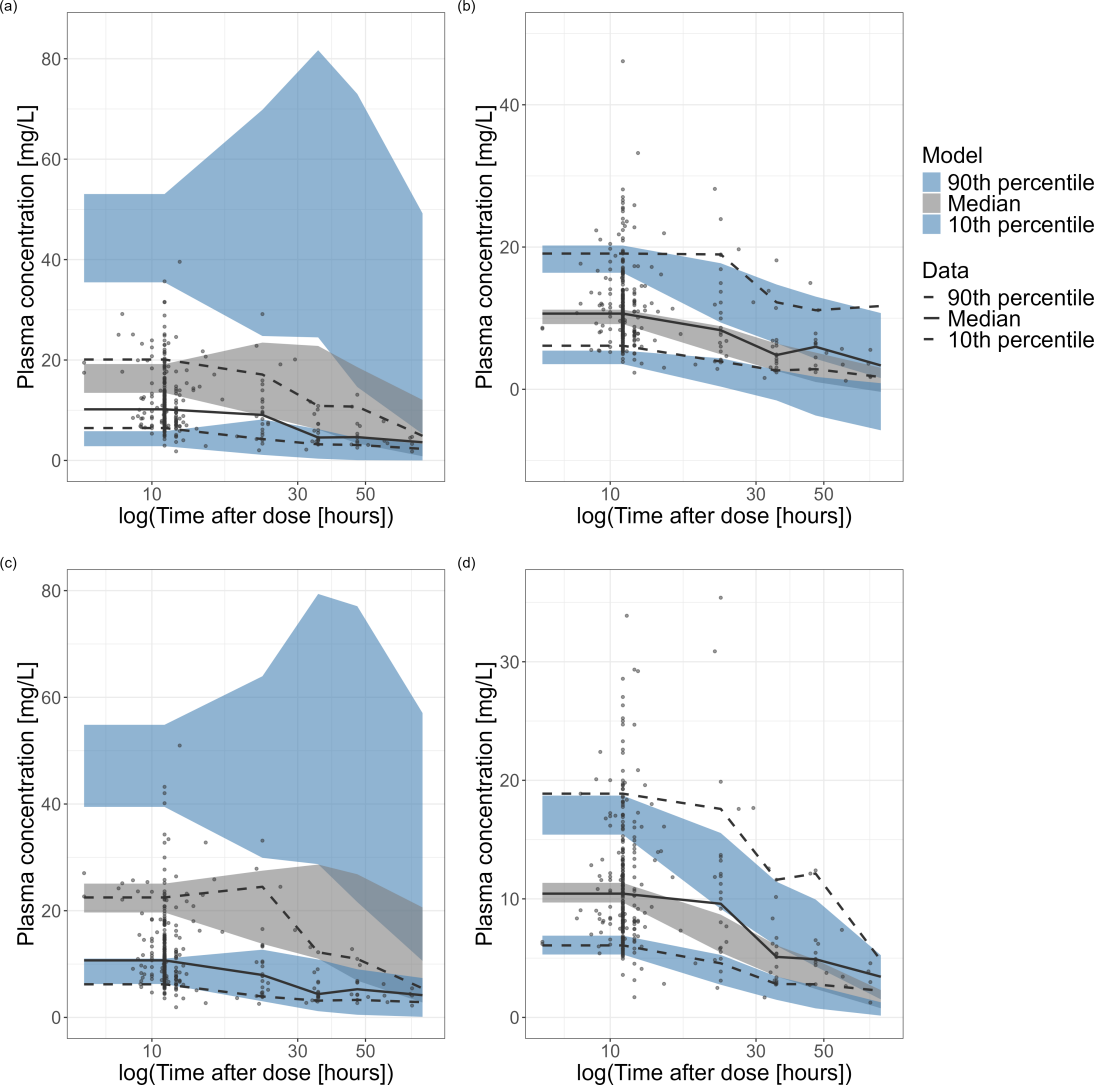
Prediction-corrected visual predictive checks (pcVPC) of the four models that were most frequently among the top five in terms of MPE and MAPE: (**a**) Bae et al.; (**b**) Bury et al.; (**c**) Garreau et al.; and (**d**) Okada et al. The black solid line and the black dashed line represent the median and the 10th/90th percentile of the data set, respectively. The gray and light blue area represents the 90% CI of the 50th and 10th/90th quantile of 1000 simulations, respectively.

In the *a priori* scenario, the MPE ranged from −199.6% to 93.3% with one model within ±10%. Thirteen models showed underprediction as reflected by negative MPE values, and eight overpredicted vancomycin concentration. The top five models in terms of MPE in this scenario were from Okada et al. (−4.0%), Bury et al. (−10.4%), Ji et al. (−12.2%), Huang et al. (13.6%), and Tanaka et al. (20.8%) (Fig. 1a). MAPE ranged from 31.6% to 199.6%, and the five best models were from Okada et al. (31.6%), Tanaka et al. (33.0%), Ji et al. (35.2%), Bury et al. (37.1%), and Huang et al. (38.9%) (Fig. 1b). The prediction-corrected visual predictive checks for further evaluation of model fit in the *a priori* scenario are shown in Fig. 2 for the top four models in terms of MPE and MAPE, and in Fig. S1 for all models.

**Fig 3:**
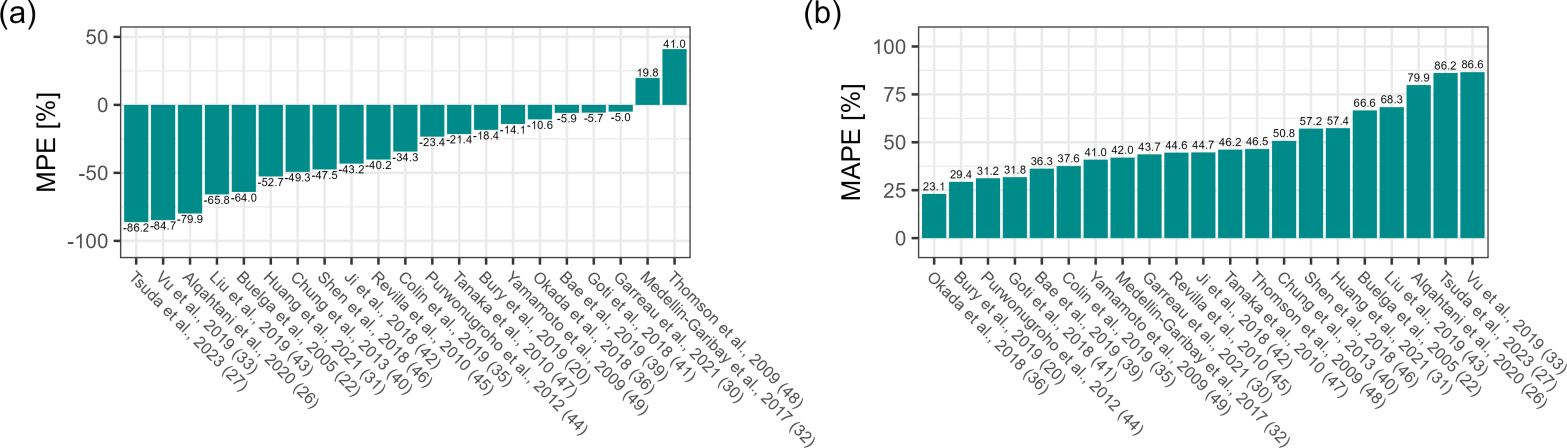
(**a**) Median prediction error (MPE [%]) and (**b**) median absolute prediction error (MAPE [%]), Bayesian scenario, first prior occasion (20, 22, 26, 27, 30–33, 35, 36, 39–49).

**Fig 4:**
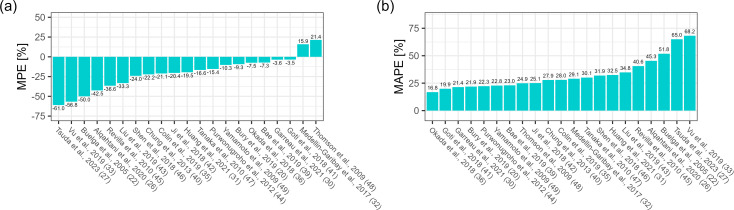
(**a**) Median prediction error (MPE [%]) and (**b**) median absolute prediction error (MAPE [%]), Bayesian scenario, second prior occasion (20, 22, 26, 27, 30–33, 35, 36, 39–49).

In the Bayesian scenario with a TDM measurement from one previous dosing occasion and covariates, the MPE ranged from −86.2% to 41.0% when using the first prior occasion and from −61.0% to 21.4% when using the second (i.e., most recent) prior occasion. There were three (first occasion) or five (second occasion) models within ±10%. In both cases, all models except those of Thomson et al. and Medellin-Garibay et al. showed underprediction. In either case, the models of Garreau et al. (first occasion −5.0%, second occasion −3.6%), Goti et al. (first occasion −5.7%, second occasion −3.5%), Bae et al. (first occasion −5.9%, second occasion −7.3%), and Okada et al. (first occasion −10.6%, second occasion −7.5%) were in the top five, together with Yamamoto et al. (−14.1%) for the first occasion scenario and Bury et al. (−9.3%) for the second occasion scenario (Fig. 3a and 4a). The MAPE ranged from 23.1% to 86.6% for the first prior occasion and from 16.8% to 68.2% when using the data from the second occasion. In the first occasion scenario, the only model below 25% MAPE was that of Okada et al. (23.1%), whereas for the scenario using the second dosing occasion, the following eight models met this condition: Okada et al. (16.8%), Goti et al. (19.9%), Garreau et al. (21.4%), Bury et al. (21.9%), Purwonugroho et al. (22.3%), Yamamoto et al. (22.8%), Bae et al. (23.0%), and Thomson et al. (24.9%). Of the top five, all but the Garreau et al. model were also among the top five in terms of MAPE in the first occasion scenario. The fifth was the Bae et al. model (36.3%), which ranked fifth after Okada et al. (23.1%), Bury et al. (29.4%), Purwonugroho et al. (31.2%), and Goti et al. (31.8%) (Fig. 3b and 4b).

**Fig 5:**
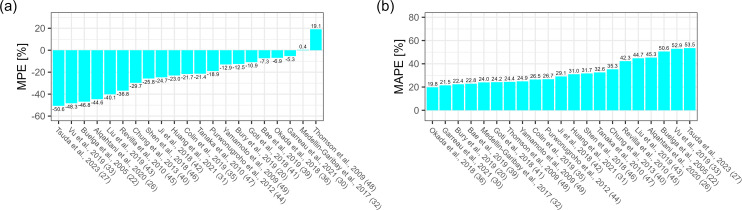
(**a**) Median prediction error (MPE [%]) and (**b**) median absolute prediction error (MAPE [%]), Bayesian scenario, two prior occasions (20, 22, 26, 27, 30–33, 35, 36, 39–49).

In the Bayesian scenario with TDM measurements from two previous dosing occasions and patient covariates, the MPE ranged from −50.6% to 19.1%, with four models within ±10%. All models, except the Thomson et al. and Medellin-Garibay et al. model, showed underprediction as reflected by negative MPE values. The five best models regarding MPE in this scenario were those of Medellin-Garibay et al. (0.4%), Garreau et al. (−5.3%), Okada et al. (−6.9%), Bae et al. (−7.3%), and Goti et al. (−10.9%) (Fig. 5a). MAPE ranged from 19.8% to 53.5%, with eight models below 25%. The top five models were Okada et al. (19.8%), Garreau et al. (21.5%), Bury et al. (22.4%), Bae et al. (22.8%), and Medellin-Garibay et al. (24.0%) (Fig. 5b).

**Fig 6:**
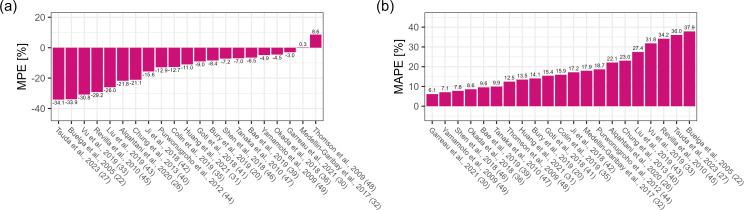
(**a**) Median prediction error (MPE [%]) and (**b**) median absolute prediction error (MAPE [%]), general model fit scenario (20, 22, 26, 27, 30–33, 35, 36, 39–49).

In the general model fit scenario, the MPE ranged from −34.1% to 8.6%, with ten models within ±10%. Consistent with the other scenarios, the Thomson et al. and Medellin-Garibay et al. models were the only ones to overpredict the vancomycin concentration. The five best models in terms of MPE in this scenario were those of Medellin-Garibay et al. (0.3%), Garreau et al. (−3.0%), Okada et al. (−4.5%), Yamamoto et al. (−4.9%), and Bae et al. (−6.5%) (Fig. 6a). MAPE ranged from 6.1% to 37.9%, with nine models below 15% and 16 below 25%. The top five models were those of Garreau et al. (6.1%), Yamamoto et al. (7.1%), Shen et al. (7.8%), Okada et al. (8.6%), and Bae et al. (9.6%) (Fig. 6b).

The model published by Okada et al. was implemented in the web-based MIPD software TDMx, as it was the model with the overall best predictive performance. It was ranked in the top five regarding MPE and MAPE in each scenario, and no major misspecifications were identified in the pcVPC.

For patients with a BMI of less than 30 (*n* = 107), there were no relevant changes in the predictive performance of the models regarding their ranking in terms of MPE and MAPE as compared to the total population. There were 22 patients with a BMI greater than or equal to 30. Some patients contributed data to both BMI groups as their weight changed during the vancomycin treatment period. In this BMI group, the Okada et al. model was still one of the most accurate and precise models, together with the following five models: Garreau et al., Tanaka et al., Revilla et al., Yamamoto et al., and Thompson et al. The Garreau et al. and Tanaka et al. models were most often in the top five for MPE and MAPE, but the Garreau et al. model was neither accurate nor precise in the *a priori* scenario (MPE and MAPE: 95%). MPE and MAPE are shown in Fig. S2 to S11 for each scenario of this sub-analysis.

## DISCUSSION

The 21 models tested vary in the size and type of the underlying population, covariates, and general model structure. Some describe the PK in patient populations that are more similar to the allo-HSCT patients included in this study than others (20, 22, 26, 27, 36). We observed many considerable differences in predictive performance and general model fit. This emphasizes the need for a population PK model that fits the population of interest well in order to apply MIPD reliably. Otherwise, the use of an inappropriate model would lead to imprecise and inaccurate predictions, potentially resulting in an inappropriate dose adjustment. Overall, predictive performance improves when not only patient covariates (*a priori*) but also one or more prior TDM measurements are considered (Bayesian forecasting and general model fit). For most models, the *a priori* scenario has the highest MPE and MAPE, reflecting the highest inaccuracy and imprecision. In the Bayesian forecasting scenario using TDM measurements from a previous dosing occasion, the predictive performance is generally improved when the most recent measurement before the reference occasion is used. In some cases, this scenario results in even better accuracy and precision than the scenario using more TDM measurements from two prior dosing occasions. These findings thus suggest the importance of using TDM measurements from the most recent prior dosing occasion in contrast to a greater quantity of earlier measurements.

Overall, the model published by Okada et al. (36) showed the best predictive performance. It was the only model to be in the top five for MPE and MAPE in every scenario. In addition, no distinct model misspecifications were observed in the pcVPC for the central tendency, except outliers at the upper percentiles and median. This indicates that the variability is overall adequately described and matches with the models’ high performance in the *a priori* scenario. Of the three Bayesian forecasting scenarios, the one using only the most recent observed concentration was the most precise, supporting the aforementioned finding. Compared to the other models tested, the Okada et al. (36) model was ranked higher in the *a priori* scenario (MPE and MAPE rank 1) than in the general model fit scenario (MPE rank 3 and MAPE rank 4). Hence, the Okada et al. (36) model appears to be the most appropriate model for *a priori* PK prediction in this allo-HSCT population, when individual patient concentrations are not available. The Okada et al. (36) model was the only model that was also developed in an allo-HSCT population. When comparing the Okada et al. (36) patient population with ours, they were slightly younger (median, range: 49 years, 17–69 vs. 58 years, 18-85), weighed less (median, range: 59.4 kg, 39.4–104.5 vs. 76.9 kg, 52.0–133.3), and had a greater proportion of male patients (65% vs. 60%). There was little difference in Cockcroft-Gault clearance between patients in the Okada et al. (36) study and those in the present study (median, range: 113 ml/min/1.73 m^2^, 47–253 vs. 103 ml/min/1.73 m^2^, 36–253). The model published by Bury et al. (20) showed the second-best accuracy after Okada et al. (36) in the *a priori* scenario. In addition, the pcVPC of this model was rated as one of the best, indicating no model misspecification. However, it was less precise than the Okada et al. (36) model with a MAPE of 37.1% in the *a priori* scenario compared to 31.6%. In the three different Bayesian forecasting scenarios, it ranked in the top five for accuracy once and for precision each time. Given that the Bury et al. (20) model was developed for patients with neutropenia associated with malignancies, it is not surprising that the model performed well in a population of patients with an allo-HSCT. They predominantly receive vancomycin to treat neutropenic fever in the aplastic phase shortly after transplantation. The vancomycin PK model developed by Garreau et al. (30) for patients receiving continuous infusion was also one of the best performing models in our model comparison. Interestingly, the predictive performance was in the top five in most of the Bayesian forecasting scenarios and the general model fit scenario, but was inadequate in the *a priori* scenario. The latter was also reflected by large visible misspecifications in the pcVPC (Fig. 2). However, it performed well in the Bayesian forecasting scenarios and general model fit. The predictive performance of the Bae et al. (39) model was similar to that of the Garreau et al. (30) model with better predictions in the Bayesian forecasting and general model fit scenario and large misspecifications in the pcVPC (Fig. 2). The suboptimal performance observed in the *a priori* scenario may be attributed to the fact that the two models were developed for a distinct population type. The Goti et al. (41) model was one of the top five models, but like the Garreau et al. (30) and Bae et al. (39) models, its predictive performance was better in the Bayesian forecasting scenarios than *a priori*. Accordingly, we have implemented the Okada et al. (36) model into the web-based MIPD software TDMx for future MIPD in allo-HSCT patients.

There were more patients with a BMI of less than 30 than with a BMI greater than or equal to 30. The Okada et al. (36) model was still the model with the best predictive performance for patients with a BMI of less than 30 and one of the top six models for patients with a BMI greater than or equal to 30. Although other models were more frequently in the top five in terms of MPE and MAPE in this BMI group, the Okada et al. (36) model was accurate and precise in all scenarios tested and outperformed most other models in the *a priori* scenario. Therefore, the Okada et al. (36) model is suitable for MIPD in all allo-HSCT patients, regardless of their BMI.

In the model evaluation study by Broeker et al. (25), the model by Goti et al. (41) performed best in a nonspecific population. The fact that this model performed well in our comparison but was inferior to others (20, 36) may be explained by the different patient populations. Broeker et al. (25) compared models for a nonspecific patient population, and the model of Goti et al. (41) was developed in such a nonspecific population, with approximately 20% of the patients receiving hemodialysis. In contrast, patients undergoing allo-HSCT are in most cases diagnosed with an underlying malignancy (51). As previously studied, vancomycin PK in allo-HSCT patients or those diagnosed with a malignant disease or within neutropenia tends to differ from the PK in nonspecific, more general populations (18–23, 52). There is one previously published comparison of different models for vancomycin PK estimation in patients undergoing allo-HSCT (53). However, they used a different methodology as their model prediction was not software-based. The model by Garreau et al. (30) was developed for continuous infusion patients and performed well for our intermittent infusion patients. This supports the finding of Heus et al. (24) that the route of administration had little impact on predictive performance. In addition, our data show that using more recent observations is preferable to using more observations, which is also supported by the results of previous model comparisons for vancomycin (24, 25).

This study has some limitations that should be acknowledged. The data collection was retrospective, including vancomycin concentrations, dosing information, and timing data. Therefore, the accuracy of the timing data may not be given in every case. Additionally, only trough concentrations from routine TDM were available, and therefore, the description of the patient PK may not be as accurate and precise as it would have been with a more comprehensive sampling method. The transferability of our results may be limited and should always be critically assessed in the context of the pharmacokinetic characteristics of the patients.

### Conclusion

In summary, many population PK models for vancomycin have been published. Some describe the PK in specific populations, such as patients with allo-HSCT, while others were developed in less specific groups. For a variety of reasons, it is not always possible, appropriate, or necessary to develop a new model for each patient population. Therefore, model comparisons such as ours are performed to find a suitable model for reliable use in MIPD. In our case, the model developed in an allo-HSCT patient population in Asia showed the best predictive performance and is the model we would recommend for MIPD in allo-HSCT patients. This highlights that a model developed in a similar population is likely to provide the best PK predictions, but we would also like to emphasize that there are models from different populations that may also show acceptable predictive performance.

## SUPPLEMENTAL MATERIAL

Supplemental material aac.00257-25-s0001.docx Tables S1 and S2; Fig. S1 to S11.

ASM does not own the copyrights to Supplemental Material that may be linked to, or accessed through, an article. The authors have granted ASM a non-exclusive, world-wide license to publish the Supplemental Material files. Please contact the corresponding author directly for reuse.
