# Supplementary material for: Towards precision dosing of vancomycin in patients with allogeneic hematopoietic stem cell transplantation: a comparison of published population pharmacokinetic models
Source: Antimicrob Agents Chemother. 2025 Aug 14;69(10):e00257-25. doi: 10.1128/aac.00257-25 (PMC12494056; doi:10.1128/aac.00257-25)
Supplement: Supplemental material — Tables S1 and S2; Fig. S1 to S11. [file aac.00257-25-s0001.docx]

**Table S1**. Main reasons for exclusion of pharmacokinetic models.

| **Title** | **Author (year)** | **Reason for exclusion** |
| --- | --- | --- |
| Plasma and interstitial fluid population pharmacokinetics of vancomycin in critically ill patients with sepsis (1) | Abraham et al. (2018) | Less than 50 patients included for model building. |
| Pharmacokinetics of vancomycin in extremely obese patients with suspected or confirmed Staphylococcus aureus infections (2) | Adane et al. (2015) | Less than 50 patients included for model building. |
| Population Pharmacokinetic Model for Vancomycin Used in Open Heart Surgery: Model-Based Evaluation of Standard Dosing Regimens (3) | Alqahtani et al. (2018) | Less than 50 patients included for model building. |
| Development of a new pharmacokinetic model for target-concentration controlled infusion of vancomycin in critically ill patients (4) | Bang et al. (2022) | Less than 50 patients included for model building. |
| Population pharmacokinetic model and dosing optimization of vancomycin in hematologic malignancies with neutropenia and augmented renal clearance (5) | Belabbas et al. (2023) | The full text was not available at our institution at the time of screening. |
| A study to explore the appropriateness of dosing regimen of vancomycin in critically ill patients in a tertiary care unit of India (6) | Belavagi et al. (2022) | Less than 50 patients included for model building. |
| A new regimen for continuous infusion of vancomycin during continuous renal replacement therapy (7) | Beumier et al. (2013) | Less than 50 patients included for model building. |
| Pharmacokinetics of Vancomycin in Elderly Patients Aged over 80 Years (8) | Bourguignon et al. (2016) | A model developed in geriatric patients was considered presumably inappropriate to describe pharmacokinetics in our patients. |
| Single-dose bone pharmacokinetics of vancomycin in a porcine implant-associated osteomyelitis model (9) | Bue et al. (2018) | For this model, the pharmacokinetics were investigated in bone, so that the model is not transferable to plasma concentrations. |
| Plasma and Cerebrospinal Fluid Population Pharmacokinetics of Vancomycin in Patients with External Ventricular Drain (10) | Chen et al. (2023) | Less than 50 patients included for model building. |
| Population Pharmacokinetics of Vancomycin in Critically Ill Adult Patients Receiving Extracorporeal Membrane Oxygenation (an ASAP ECMO Study) (11) | Cheng et al. (2022) | Less than 50 patients included for model building. |
| Population Pharmacokinetics of Vancomycin in Patients Receiving Hemodialysis in a Malian and a French Center and Simulation of the Optimal Loading Dose (12) | Coulibaly et al. (2023) | The full text was not available at our institution at the time of screening. |
| Initial dosage regimens of vancomycin for Chinese adult patients based on population pharmacokinetic analysis (13) | Deng et al. (2013) | The full text was not available at our institution at the time of screening. |
| Vancomycin pharmacokinetics in patients with severe burn injuries (14) | Dolton et al. (2010) | A model developed in burn patients was considered presumably inappropriate to describe pharmacokinetics in our patients. |
| Vancomycin population pharmacokinetics during extracorporeal membrane oxygenation therapy: a matched cohort study (15) | Donadello et al. (2014) | Less than 50 patients included for model building. |
| Optimizing Vancomycin Dosing in Chronic Kidney Disease by Deriving and Implementing a Web-Based Tool Using a Population Pharmacokinetics Analysis (16) | Dorajoo et al. (2019) | The model could not be recoded, as mandatory information regarding clearance calculation was missing. |
| Population pharmacokinetics of vancomycin in critically ill patients receiving prolonged intermittent renal replacement therapy (17) | Economou et al. (2018) | Less than 50 patients included for model building. |
| Population pharmacokinetics and dose simulation of vancomycin in critically ill patients during high-volume haemofiltration (18) | Escobar et al. (2014) | Less than 50 patients included for model building. |
| Clinical application of vancomycin population pharmacokinetics model in patients with hematological diseases and neutropenia (19) | Fu et al. (2021) | The model could not be recoded, as mandatory information regarding the error model was missing. |
| Influence of renal function estimation on pharmacokinetic modeling of vancomycin in elderly patients (20) | Glatard et al. (2015) | The model could not be recoded as a non-parametric approach was used to develop the model. |
| Vancomycin serum concentrations do not adequately predict tissue exposure in diabetic patients with mild to moderate limb infections (21) | Hamada et al. (2015) | Less than 50 patients included for model building. |
| Intraperitoneally Administered Vancomycin in Patients with Peritoneal Dialysis-Associated Peritonitis: Population Pharmacokinetics and Dosing Implications (22) | Hartinger et al. (2023) | Less than 50 patients included for model building. |
| Population pharmacokinetics of vancomycin and prediction of pharmacodynamics in the Chinese people (23) | He et al. (2014) | The full text was not available at our institution at the time of screening. |
| Population Pharmacokinetics and Dosing Optimization of Vancomycin in Infants, Children, and Adolescents with Augmented Renal Clearance (24) | He et al. (2021) | A model predominantly developed in non-adult patients was considered presumably inappropriate to describe pharmacokinetics in our patients. |
| Vancomycin population pharmacokinetics for adult patients with sepsis or septic shock: are current dosing regimens sufficient? (25) | Heffernan et al. (2019) | Less than 50 patients included for model building. |
| Optimizing vancomycin dosage regimens in relation to high-flux haemodialysis (26) | Hui et al. (2019) | Less than 50 patients included for model building. |
| Population Pharmacokinetic Modeling of Vancomycin in Thai Patients With Heterogeneous and Unstable Renal Function (27) | Jaisue et al. (2020) | The model could not be recoded because the baseline clearance could not be adequately incorporated. |
| Development and comparison of population pharmacokinetic models of vancomycin in neurosurgical patients based on two different renal function markers (28) | Jing et al. (2020) | A model developed in surgical patients was considered presumably inappropriate to describe pharmacokinetics in our patients. |
| Prospective Cohort Study of Population Pharmacokinetics and Pharmacodynamic Target Attainment of Vancomycin in Adults on Extracorporeal Membrane Oxygenation (29) | Jung et al. (2021) | Less than 50 patients included for model building. |
| Vancomycin Population Pharmacokinetics in Critically Ill Adults During Sustained Low-Efficiency Dialysis (30) | Kanji et al. (2020) | Less than 50 patients included for model building. |
| Comparison of the pharmacokinetics of vancomycin in neurosurgical and non-neurosurgical patients (31) | Kim et al. (2016) | The full text was not available at our institution at the time of screening. |
| A new population pharmacokinetic model for vancomycin in patients with variable renal function: Therapeutic drug monitoring based on extended covariate model using CKD-EPI estimation (32) | Kim et al. (2019) | The model could not be recoded because the time-dependent clearance could not be adequately incorporated. |
| Exploring population pharmacokinetic models in patients treated with vancomycin during continuous venovenous haemodiafiltration (CVVHDF) (33) | Kirwan et al. (2021) | Less than 50 patients included for model building. |
| Population pharmacokinetic model of Vancomycin based on therapeutic drug monitoring data in critically ill septic patients (34) | Kovacevic et al. (2020) | A model developed in septic patients was considered presumably inappropriate to describe pharmacokinetics in our patients. |
| Plasma and cerebrospinal fluid population pharmacokinetics of vancomycin in postoperative neurosurgical patients after combined intravenous and intraventricular administration (35) | Li et al. (2017) | Less than 50 patients included for model building. |
| Population Pharmacokinetics of Vancomycin in Postoperative Neurosurgical Patients and the Application in Dosing Recommendation (36) | Li et al. (2016) | The full text was not available at our institution at the time of screening. |
| Population Pharmacokinetics of Vancomycin in Postoperative Neurosurgical Patients (37) | Li et al. (2015) | Less than 50 patients included for model building. |
| Population pharmacokinetics of vancomycin in adult Chinese patients with post-craniotomy meningitis and its application in individualised dosage regimens (38) | Lin et al. (2016) | A model developed in surgical patients was considered presumably inappropriate to describe pharmacokinetics in our patients. |
| Population pharmacokinetic modeling and clinical application of vancomycin in Chinese patients hospitalized in intensive care units (39) | Lin et al. (2021) | The model could not be recoded, as mandatory information regarding the error model was missing. |
| Population pharmacokinetic parameters of vancomycin in critically ill patients (40) | Llopis-Salvia et al. (2006) | Less than 50 patients included for model building. |
| Population Pharmacokinetics of Vancomycin in Kidney Transplant Recipients: Model Building and Parameter Optimization (41) | Ma et al. (2020) | A model developed in kidney transplant patients was considered presumably inappropriate to describe pharmacokinetics in our patients. |
| Vancomycin pharmacokinetic and pharmacodynamic models for critically ill patients with post-sternotomy mediastinitis (42) | Mangin et al. (2014) | Less than 50 patients included for model building. |
| Pharmacokinetics of vancomycin and dosing recommendations for trauma patients (43) | Medellin-Garibay et al. (2016) | A model developed in trauma patients was considered presumably inappropriate to describe pharmacokinetics in our patients. |
| Vancomycin pharmacokinetics during continuous ambulatory peritoneal dialysis in patients with peritonitis (44) | Montanes Pauls et al. (2011) | Less than 50 patients included for model building. |
| A Population Pharmacokinetic Model for Vancomycin in Adult Patients Receiving Extracorporeal Membrane Oxygenation Therapy (45) | Moore et al. (2016) | Less than 50 patients included for model building. |
| Population pharmacokinetics of vancomycin in patients receiving extracorporeal membrane oxygenation (46) | Mulla et al. (2005) | Less than 50 patients included for model building. |
| Dose Tailoring of Vancomycin Through Population Pharmacokinetic Modeling Among Surgical Patients in Pakistan (47) | Munir et al. (2021) | A model developed in surgical patients was considered presumably inappropriate to describe pharmacokinetics in our patients. |
| Development of Vancomycin Dose Individualization Strategy by Bayesian Prediction in Patients Receiving Continuous Renal Replacement Therapy (48) | Oda et al. (2020) | Less than 50 patients included for model building. |
| Model-Informed Precision Dosing of Vancomycin in Adult Patients Undergoing Hemodialysis (49) | Oda et al. (2023) | Less than 50 patients included for model building. |
| Pharmacokinetics of Vancomycin among Patients with Chemotherapy-Associated Febrile Neutropenia: Which Would Be the Best Dosing to Obtain Appropriate Exposure? (50) | Parra Gonzalez et al. (2022) | Less than 50 patients included for model building. |
| Development of a Physiologically Based Pharmacokinetic Modelling Approach to Predict the Pharmacokinetics of Vancomycin in Critically Ill Septic Patients (51) | Radke et al. (2017) | A model developed in septic patients was considered presumably inappropriate to describe pharmacokinetics in our patients. |
| Individualized Delivery of Vancomycin by Model-Informed Bayesian Dosing Approach to Maintain an AUC24 Target in Critically Ill Patients (52) | Rao et al. (2024) | The full text was not available at our institution at the time of screening. |
| Vancomycin dosing in critically ill patients: robust methods for improved continuous-infusion regimens (53) | Roberts et al. (2011) | A model developed in septic patients was considered presumably inappropriate to describe pharmacokinetics in our patients. |
| Population pharmacokinetics of vancomycin in adult and geriatric patients: comparison of eleven approaches (54) | Sanchez et al. (2010) | The full text was not available at our institution at the time of screening. |
| A Bayesian Model to Describe Factors Influencing Trough Levels of Vancomycin in Hemodialysis Patients (55) | Sansot et al. (2015) | The full text was not available at our institution at the time of screening. |
| Urea kinetics and dialysis treatment time predict vancomycin elimination during high-flux hemodialysis (56) | Schaedeli et al. (1998) | Less than 50 patients included for model building. |
| Comparison of Race-Based and Non-Race-Based Equations for Kidney Function Estimation in Critically Ill Thai Patients for Vancomycin Dosing (57) | Sitaruno et al. (2022) | The model could not be recoded because of the lack of mandatory information on the units which have been used. |
| Population pharmacokinetics of vancomycin in obesity: Finding the optimal dose for (morbidly) obese individuals (58) | Smit et al. (2020) | Less than 50 patients included for model building. |
| Optimised dosing of vancomycin in critically ill Indigenous Australian patients with severe sepsis (59) | Tsai et al. (2018) | Less than 50 patients included for model building. |
| Can population pharmacokinetic modelling guide vancomycin dosing during continuous renal replacement therapy in critically ill patients? (60) | Udy et al. (2013) | A model developed in patients with continuous renal replacement therapy was considered presumably inappropriate to describe pharmacokinetics in our patients. |
| Therapeutic drug monitoring of vancomycin in severe sepsis and septic shock (61) | Vazquez et al. (2008) | The full text was not available at our institution at the time of screening. |
| Determination of vancomycin exposure target and individualized dosing recommendations for critically ill patients undergoing continuous renal replacement therapy (62) | Wang et al. (2023) | A model developed in patients with continuous renal replacement therapy was considered presumably inappropriate to describe pharmacokinetics in our patients. |
| Dose Optimization of Vancomycin for Critically Ill Patients Undergoing CVVH: A Prospective Population PK/PD Analysis (63) | Wang et al. (2021) | Less than 50 patients included for model building. |
| Population pharmacokinetic model of vancomycin in postoperative neurosurgical patients (64) | Wei et al. (2022) | A model developed in surgical patients was considered presumably inappropriate to describe pharmacokinetics in our patients. |
| Vancomycin pharmacokinetic model development in patients on intermittent online hemodiafiltration (65) | Westra et al. (2019) | Less than 50 patients included for model building. |
| Pharmacokinetics of vancomycin in adults receiving extracorporeal membrane oxygenation (66) | Wu et al. (2016) | Less than 50 patients included for model building. |
| Establishment and application of population pharmacokinetics model of vancomycin in infants with meningitis (67) | Xu et al. (2022) | A model developed in infants was considered presumably inappropriate to describe pharmacokinetics in our patients. |
| Population pharmacokinetics and target attainment analysis of vancomycin after intermittent dosing in adults with cystic fibrosis (68) | Yellepeddi et al. (2024) | Less than 50 patients included for model building. |
| Population pharmacokinetics and individualized dosing of vancomycin for critically ill patients receiving continuous renal replacement therapy: the role of residual diuresis (69) | Yu et al. (2023) | A model developed in patients with continuous renal replacement therapy was considered presumably inappropriate to describe pharmacokinetics in our patients. |
| Pharmacokinetics of Vancomycin in Patients with Different Renal Function Levels (70) | Zaric et al. (2018) | Less than 50 patients included for model building. |
| Population Pharmacokinetic Modeling and Dose Optimization of Vancomycin in Chinese Patients with Augmented Renal Clearance (71) | Zhao et al. (2021) | The model could not be recoded, as mandatory information regarding the error model was missing. |
| Development of a Population Pharmacokinetic Model of Vancomycin and its Application in Chinese Geriatric Patients with Pulmonary Infections (72) | Zhou et al. (2019) | A model developed in geriatric patients was considered presumably inappropriate to describe pharmacokinetics in our patients. |

**Table S2**. Characteristics describing the 21 pharmacokinetic models.

| **Title** | **Author (year)** | **Number of patients** | **Number of CMT** | **Covariates** | | | | **Comment** |
| --- | --- | --- | --- | --- | --- | --- | --- | --- |
|  | | | | **CL** | **V1** | **V2** | **Q** |  |
| Optimization of Vancomycin Dosing Regimen in Cancer Patients using Pharmacokinetic/Pharmacodynamic Modeling (73) | Alqahtani et al. (2020) | 147 | 1 | Creatinine clearance (Cockcroft and Gault equation) | / | / | / | The IIV was implemented exponentially, although it was reported as proportional in the publication, as otherwise the NONMEM run failed. Serum creatinine was reported in mmol/l. As the values given would not be plausible with this unit, they were assumed to be in µmol/l. |
| Application of Pharmacometrics in Pharmacotherapy: Open-Source Software for Vancomycin Therapeutic Drug Management (74) | Bae et al. (2019) | 220 | 2 | Creatinine clearance (Cockcroft and Gault equation) | / | Total body weight | / | Dialysis status was set to 0 (no dialysis) for each individual. |
| Population pharmacokinetic analysis of vancomycin in patients with hematological malignancies (75) | Buelga et al. (2005) | 215 | 1 | Creatinine clearance (Cockcroft and Gault equation) | Total body weight | / | / | / |
| The effect of neutropenia on the clinical pharmacokinetics of vancomycin in adults (76) | Bury et al. (2019) | 116 | 2 | Creatinine clearance (Cockcroft and Gault equation), Presence of neutropenia | Fat-free mass | Fat-free mass | Fat-free mass | In the publication, neutropenia was defined as an absolute neutrophil count below 1.5 billion/l. In our dataset, neutropenia was assumed when the leucocyte count was below 1 billion/l. |
| Serum cystatin C is a major predictor of vancomycin clearance in a population pharmacokinetic analysis of patients with normal serum creatinine concentrations (77) | Chung et al. (2013) | 678 | 1 | Age, sex, total body weight, serum cystatin C, serum creatinine | Age, sex, total body weight | / | / | / |

*CMT = compartment, CL = clearance, V1 = central volume of distribution, V2 = peripheral volume of distribution, Q = intercompartmental clearance

| **Title** | **Author (year)** | **Number of patients** | **Number of CMT** | **Covariates** | | | | **Comment** |
| --- | --- | --- | --- | --- | --- | --- | --- | --- |
|  | | | | **CL** | **V1** | **V2** | **Q** |  |
| Vancomycin Pharmacokinetics Throughout Life: Results from a Pooled Population Analysis and Evaluation of Current Dosing Recommendations (78) | Colin et al. (2019) | 2554 | 2 | Postmenstrual age, serum creatinine, central volume of distribution, hematological disease | Total body weight, use of heel prick samples | Total body weight | Peripheral volume of distribution, use of heel prick samples | / |
| Population Pharmacokinetics and Dosing Simulation of Vancomycin Administered by Continuous Injection in Critically Ill Patient (79) | Garreau et al. (2021) | 78 | 2 | Creatinine clearance (Cockcroft and Gault equation) | Ideal body weight | / | / | / |
| Hospitalized Patients With and Without Hemodialysis Have Markedly Different Vancomycin Pharmacokinetics: A Population Pharmacokinetic Model-Based Analysis (80) | Goti et al. (2018) | 1812 | 2 | Creatinine clearance (Cockcroft and Gault equation), dialysis status | Total body weight, dialysis status | / | / | / |
| Cystatin C and/or creatinine-based estimated glomerular filtration rate for prediction of vancomycin clearance in long-stay critically ill patients with persistent inflammation, immunosuppression and catabolism syndrome (PICS): a population pharmacokinetics analysis (81) | Huang et al. (2021) | 69 | 1 | Glomerular filtration rate (combined CKD-EPI equation) | /* | / | / | *Volume of distribution is not estimated but calculated based on age and total body weight. |

*CMT = compartment, CL = clearance, V1 = central volume of distribution, V2 = peripheral volume of distribution, Q = intercompartmental clearance

| **Title** | **Author (year)** | **Number of patients** | **Number of CMT** | **Covariates** | | | | **Comment** |
| --- | --- | --- | --- | --- | --- | --- | --- | --- |
|  | | | | **CL** | **V1** | **V2** | **Q** |  |
| Influences of renal function descriptors on population pharmacokinetic modeling of vancomycin in Chinese adult patients (82) | Ji et al. (2018) | 160 | 1 | Creatinine clearance (Cockcroft and Gault equation), age | / | / | / | / |
| A population pharmacokinetic model of vancomycin for dose individualization based on serum cystatin C as a marker of renal function (83) | Liu et al. (2019) | 200 | 1 | Glomerular filtration rate (Hoek equation), age, total body weight | / | / | / | / |
| Influence of Mechanical Ventilation on the Pharmacokinetics of Vancomycin Administered by Continuous Infusion in Critically Ill Patients (84) | Medellin-Garibay et al. (2017) | 54 | 1 | Creatinine clearance (Cockcroft and Gault equation), presence of mechanical ventilation | Total body weight | / | / | Presence of mechanical ventilation was set to 0 (no mechanical ventilation) for each individual. |
| Population Pharmacokinetics of Vancomycin in Patients Undergoing Allogeneic Hematopoietic Stem-Cell Transplantation (85) | Okada et al. (2018) | 95 | 2 | Creatinine clearance (Cockcroft and Gault equation) | Total body weight | / | / | / |
| Population pharmacokinetics of vancomycin in Thai patients (86) | Purwonugroho et al. (2012) | 212 | 2 | Creatinine clearance (Cockcroft and Gault equation) | Age | / | / | / |

*CMT = compartment, CL = clearance, V1 = central volume of distribution, V2 = peripheral volume of distribution, Q = intercompartmental clearance

| **Title** | **Author (year)** | **Number of patients** | **Number of CMT** | **Covariates** | | | | **Comment** |
| --- | --- | --- | --- | --- | --- | --- | --- | --- |
|  | | | | **CL** | **V1** | **V2** | **Q** |  |
| Vancomycin dosing assessment in intensive care unit patients based on a population pharmacokinetic/pharmacodynamic simulation (87) | Revilla et al. (2010) | 191 | 1 | Glomerular filtration rate (Levey equation), total body weight, age | Total body weight, serum creatinine above 1mg/dl | / | / | / |
| Model-based Evaluation of the Clinical and Microbiological Efficacy of Vancomycin: A Prospective Study of Chinese Adult In-house Patients (88) | Shen et al. (2018) | 402 | 1 | Creatinine clearance (Cockcroft and Gault equation*) | Age | / | / | *No specific information on the method used for creatinine clearance determination available in the original publication. Therefore, we calculated it with the commonly used Cockcroft and Gault equation. |
| Population pharmacokinetic analysis of vancomycin using serum cystatin C as a marker of renal function (89) | Tanaka et al. (2010) | 86 | 1 | Glomerular filtration rate (Hoek equation) | Total body weight | / | / | / |
| Development and evaluation of vancomycin dosage guidelines designed to achieve new target concentrations (90) | Thomson et al. (2009) | 398 | 2 | Creatinine clearance (Cockcroft and Gault equation) | Total body weight | Total body weight | / | / |
| Population Pharmacokinetic Analysis of Vancomycin in Patients with Solid or Hematological Malignancy in Relation to the Quick Sequential Organ Failure Assessment Scores (91) | Tsuda et al. (2023) | 325 | 1 | Creatinine clearance (Cockcroft and Gault equation) | /* | / | / | *Volume of distribution is not estimated but calculated based on age and total body weight. |

*CMT = compartment, CL = clearance, V1 = central volume of distribution, V2 = peripheral volume of distribution, Q = intercompartmental clearance

| **Title** | **Author (year)** | **Number of patients** | **Number of CMT** | **Covariates** | | | | **Comment** |
| --- | --- | --- | --- | --- | --- | --- | --- | --- |
|  | | | | **CL** | **V1** | **V2** | **Q** |  |
| Determination of optimal loading and maintenance doses for continuous infusion of vancomycin in critically ill patients: Population pharmacokinetic modelling and simulations for improved dosing schemes (92) | Vu et al. (2019) | 55 | 2 | Creatinine clearance (Cockcroft and Gault equation) | Total body weight | Total body weight | / | / |
| Population pharmacokinetic analysis of vancomycin in patients with gram-positive infections and the influence of infectious disease type (93) | Yamamoto et al. (2009) | 100 | 2 | Creatinine clearance (Cockcroft and Gault equation) | Total body weight | / | / | / |

*CMT = compartment, CL = clearance, V1 = central volume of distribution, V2 = peripheral volume of distribution, Q = intercompartmental clearance


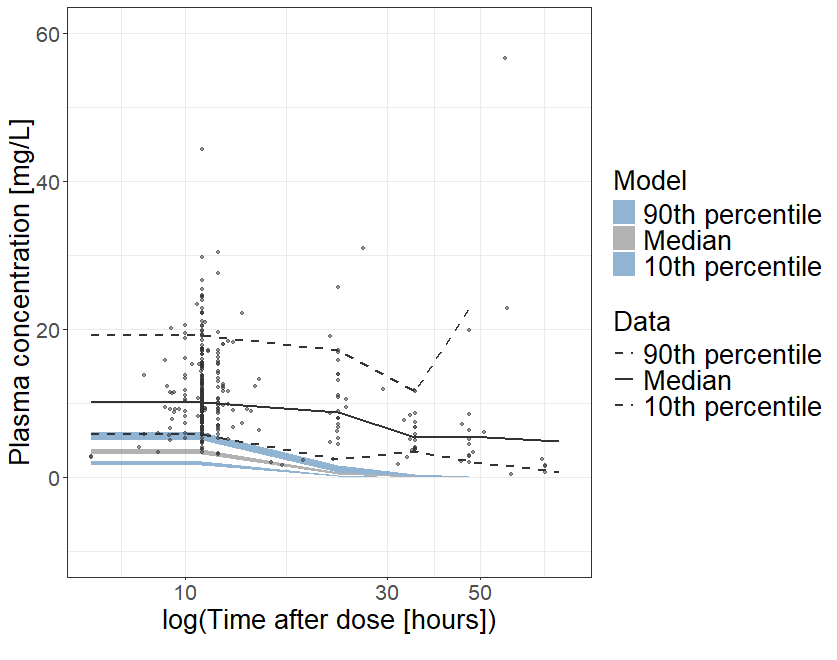

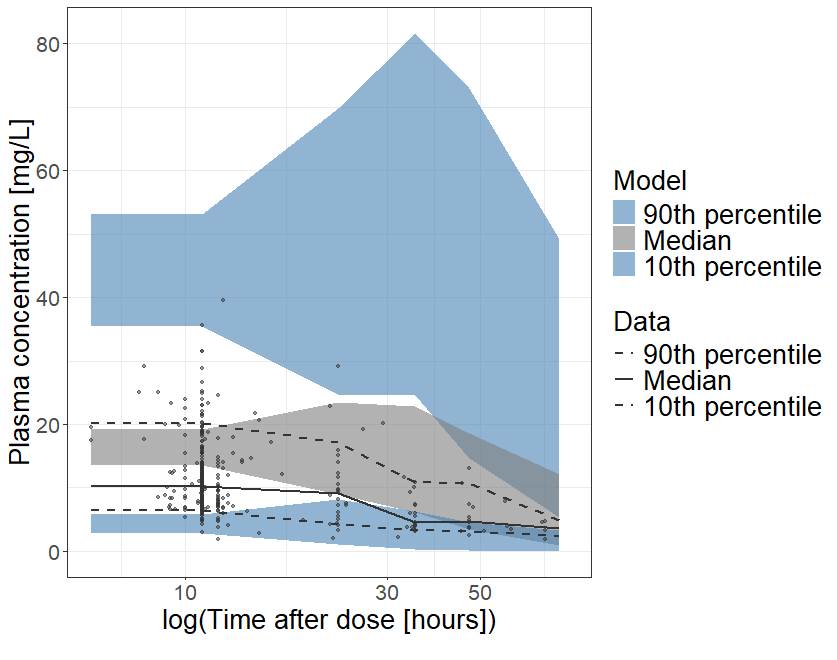


(a) (b)


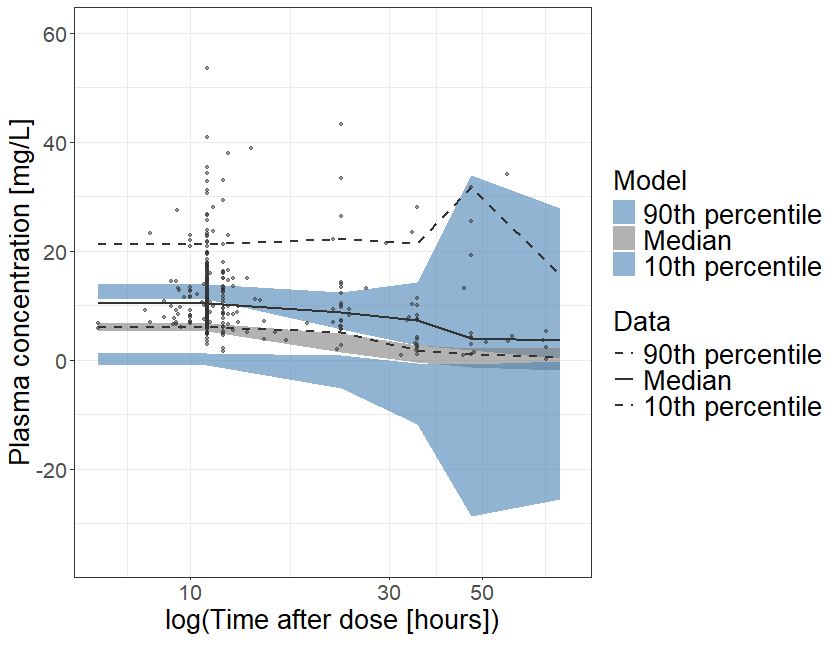

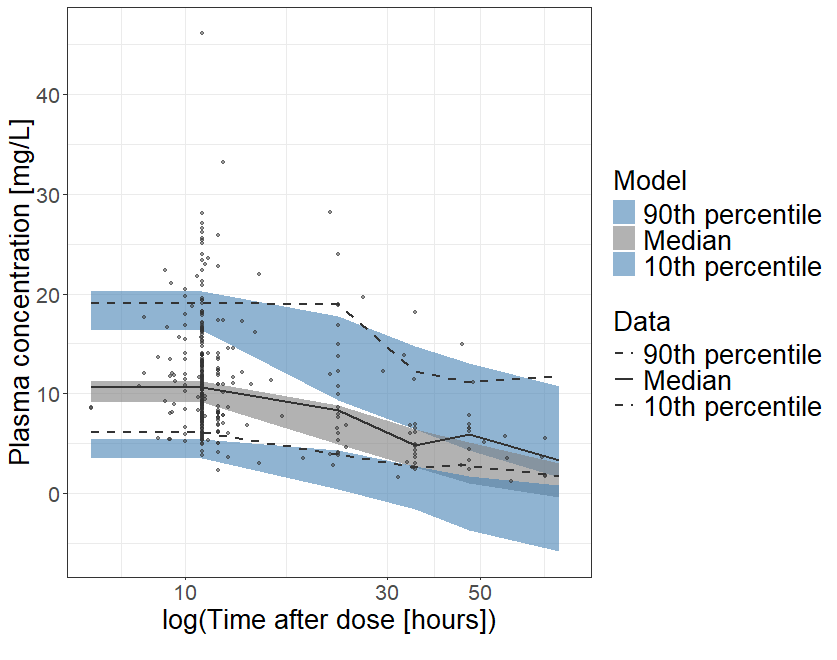


(c) (d)


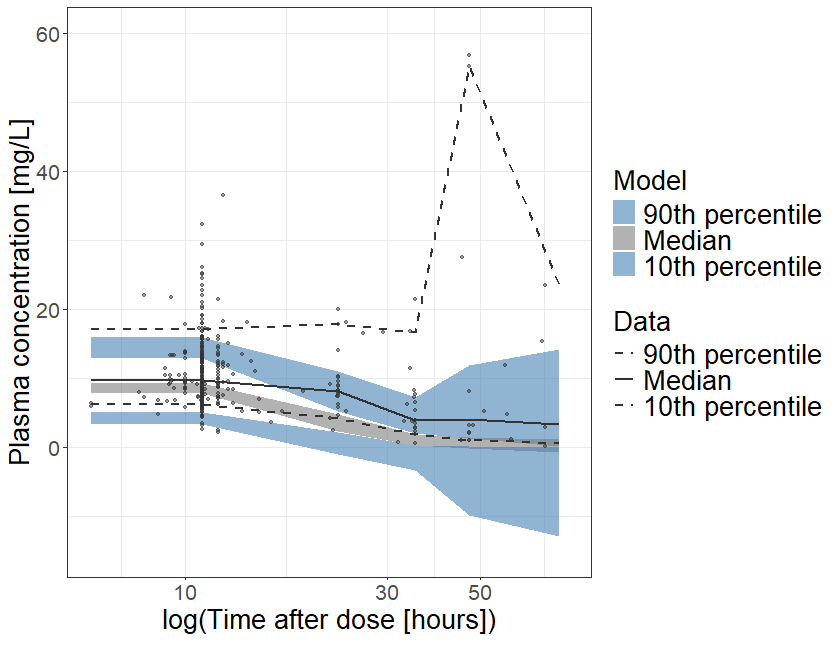

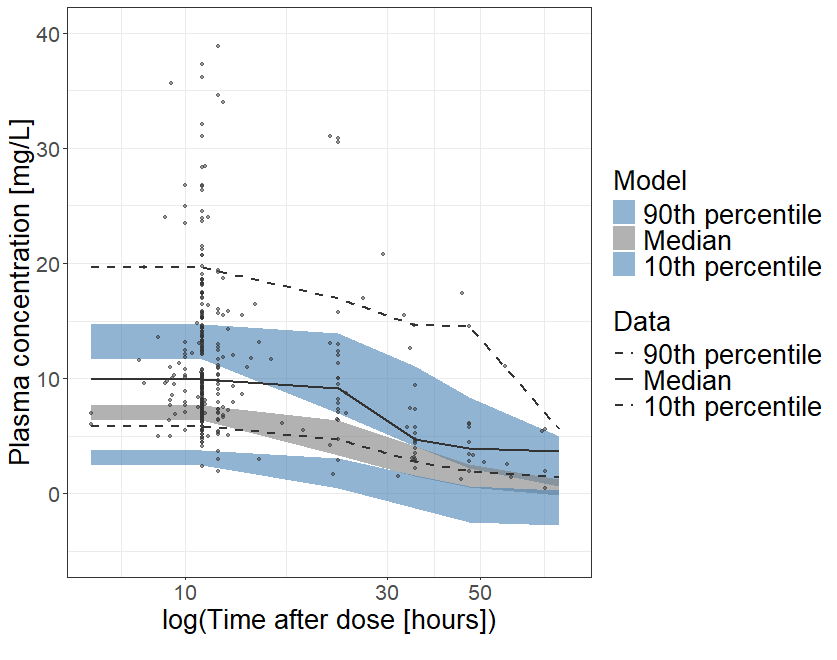


(e) (f)


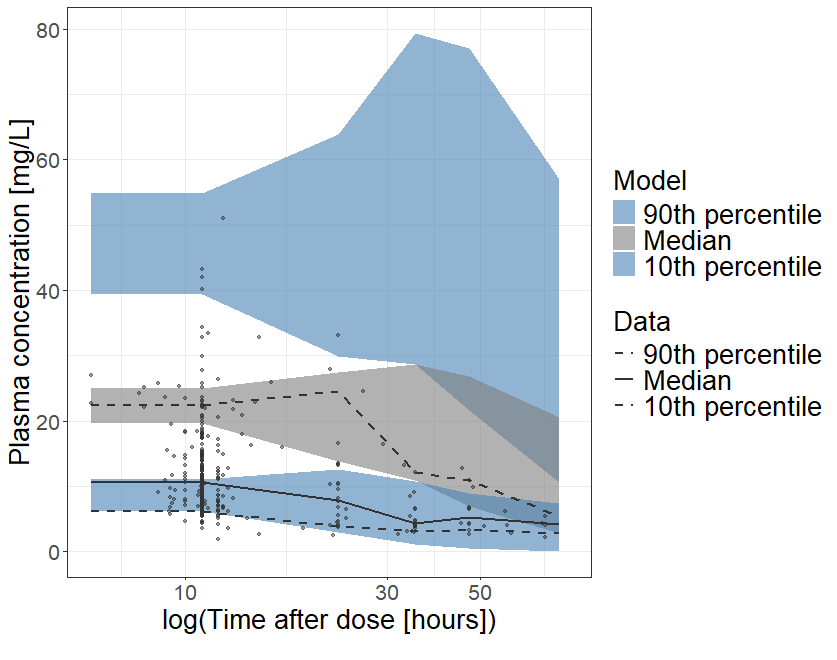

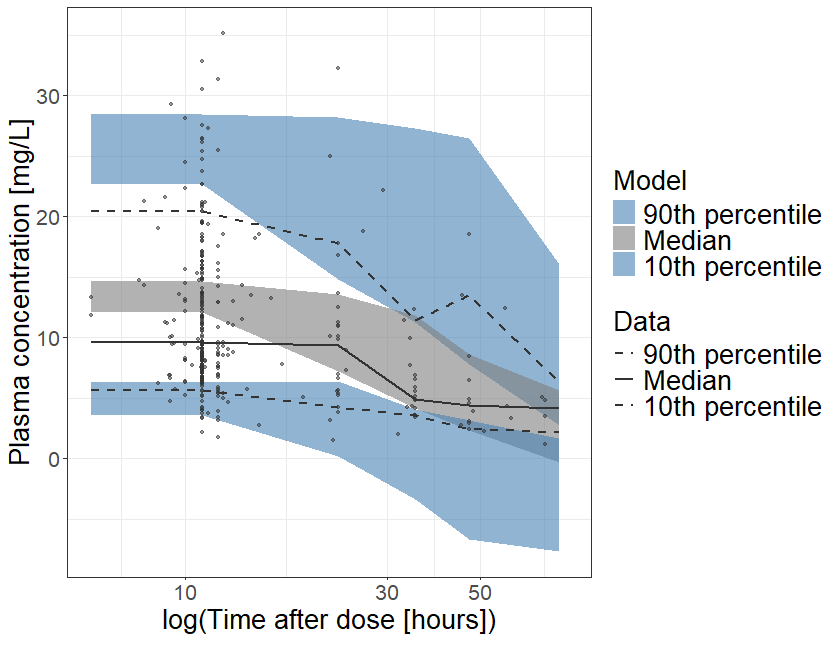


(g) (h)


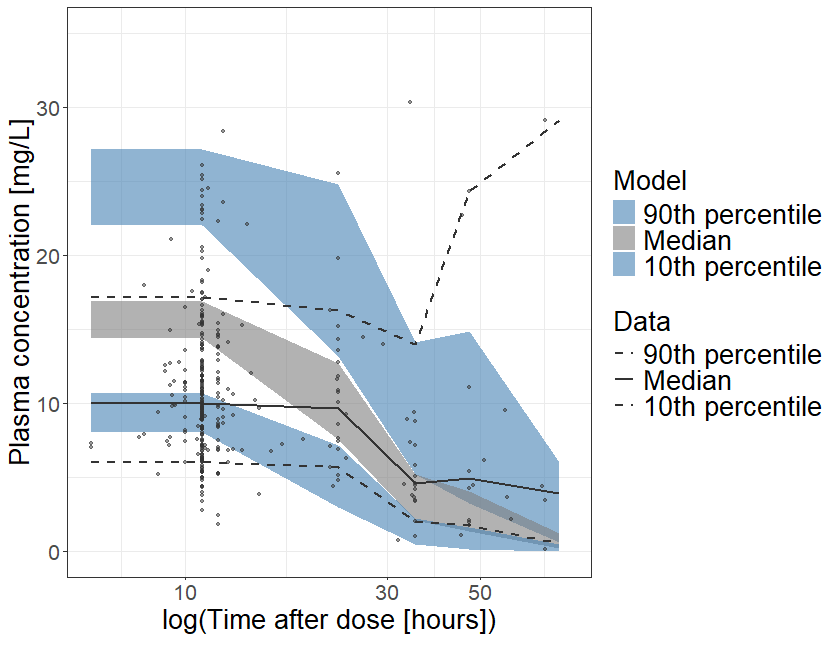

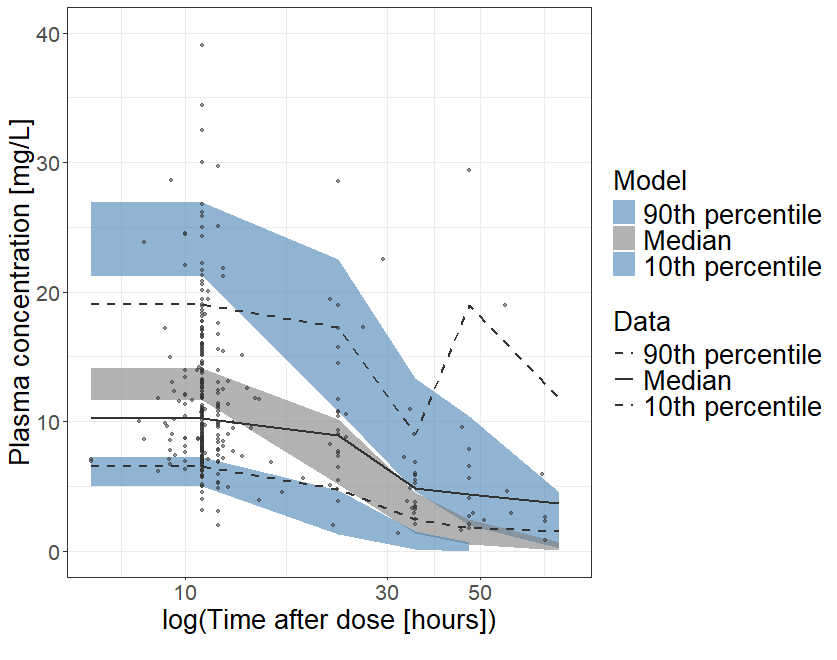


1. (j)


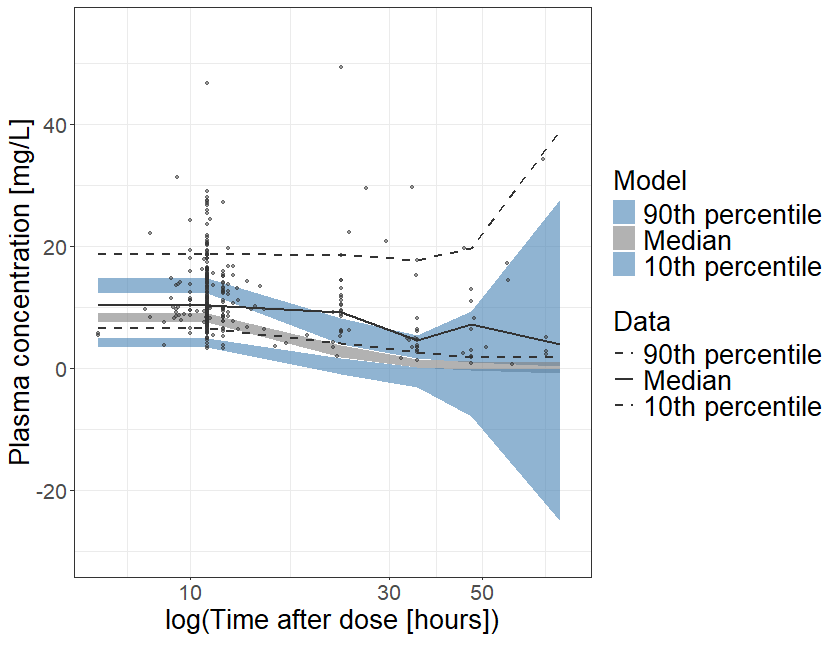

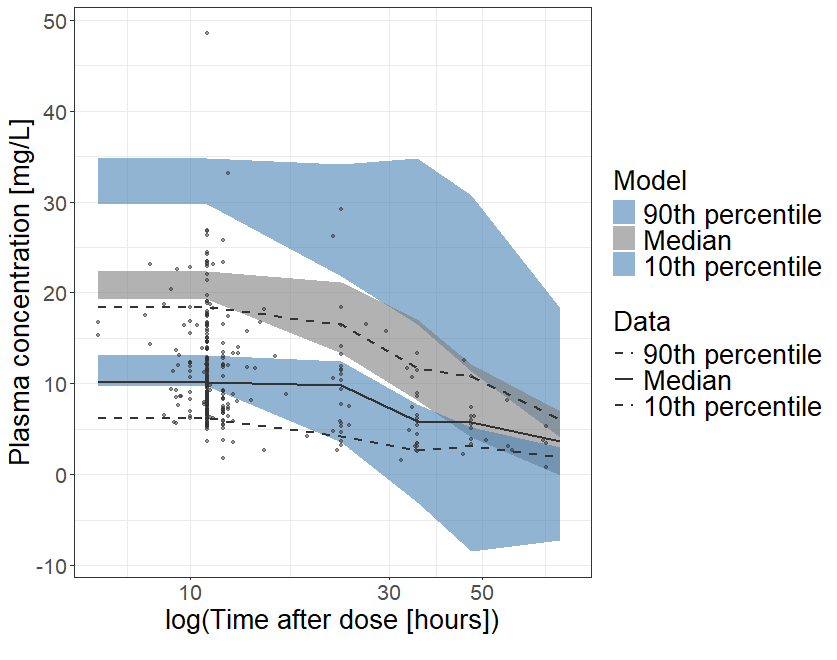


(k) (l)


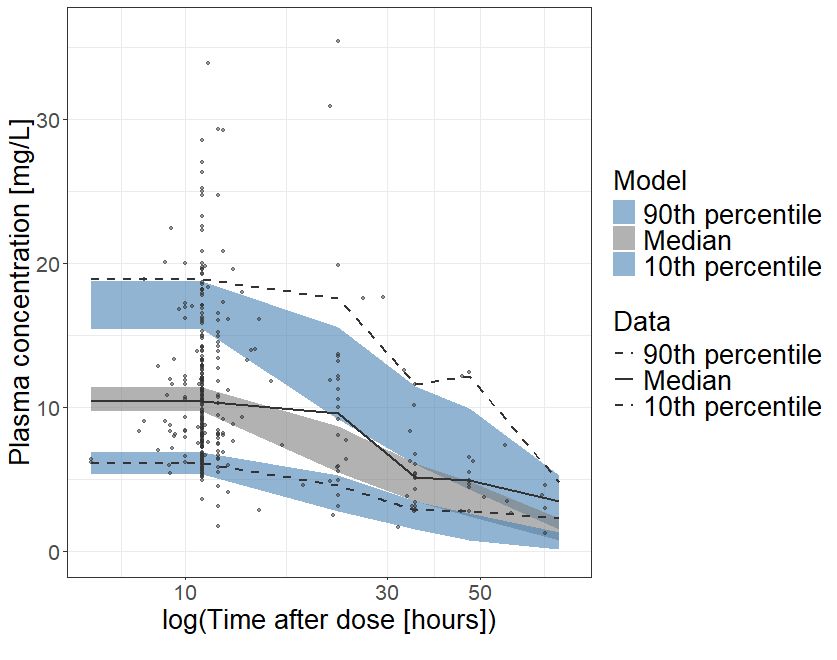

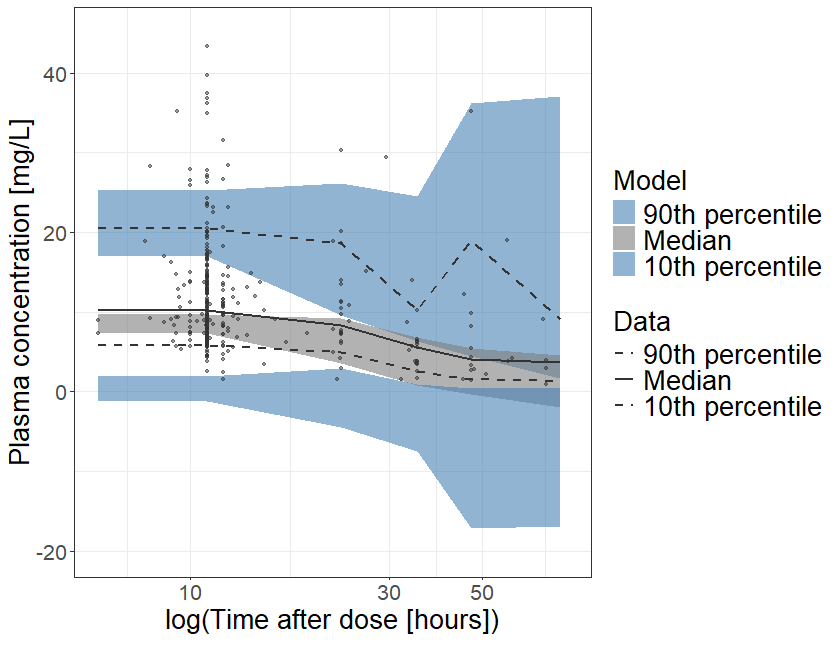


(m) (n)


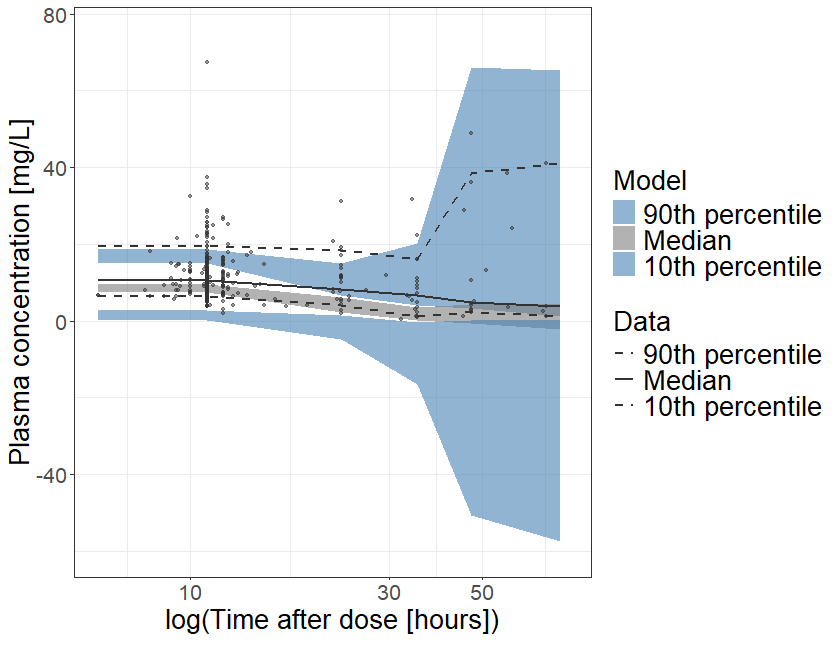

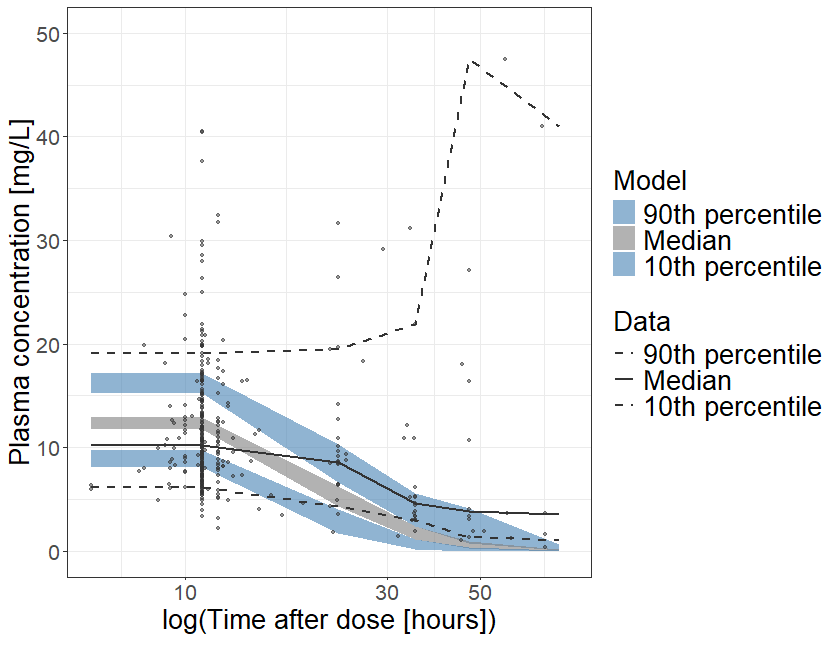


(o) (p)


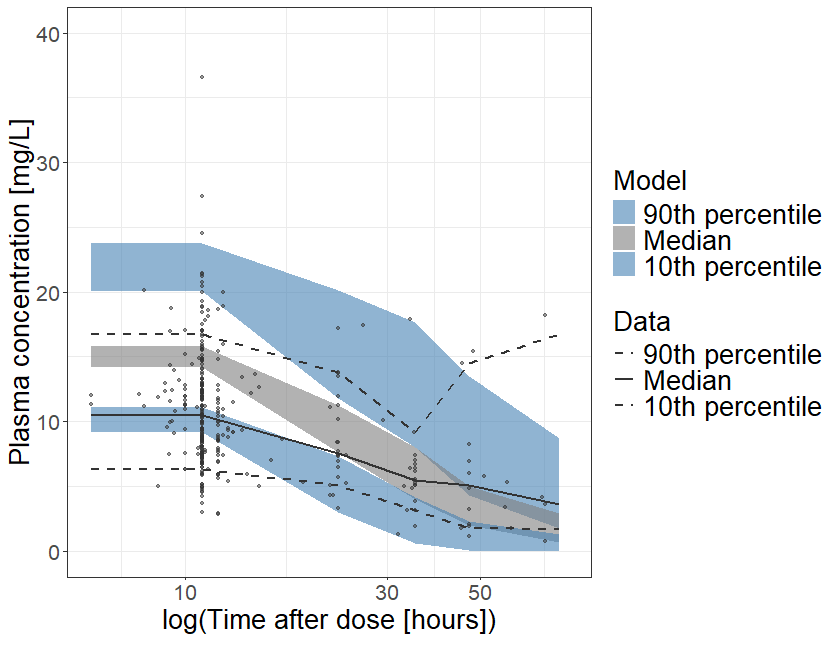

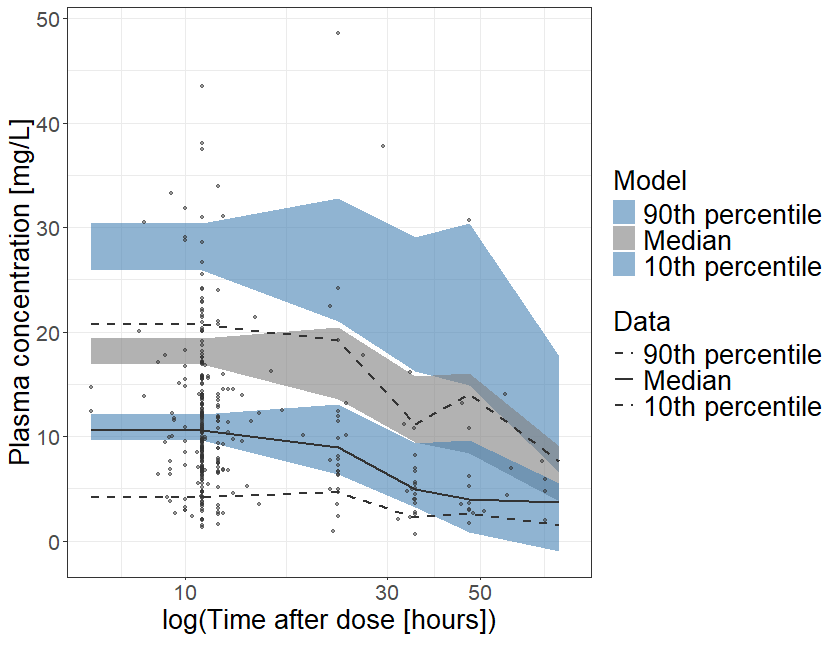


(q) (r)


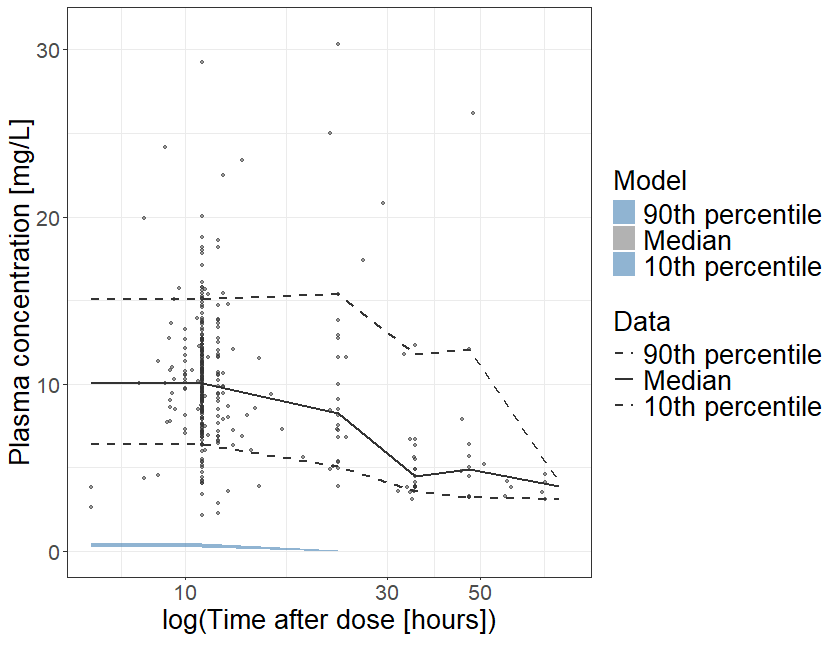

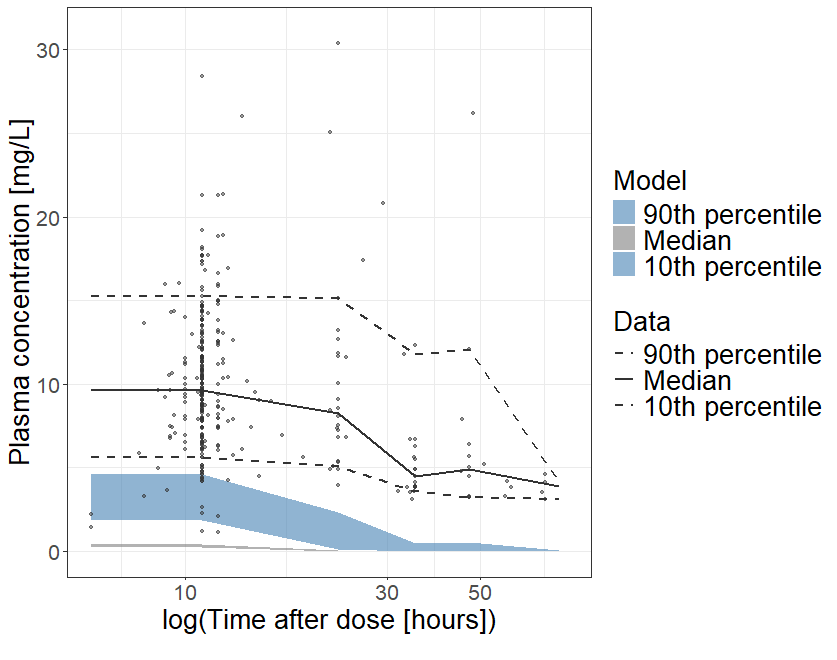


(s) (t)


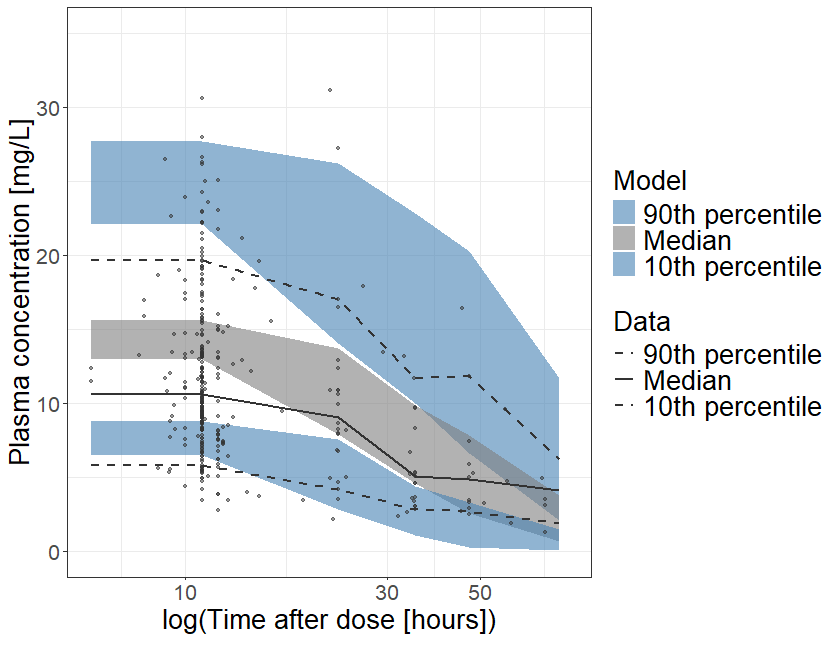


(u)

**Figure S1**. Prediction corrected visual predictive checks of the 21 PK models: (a) Alqahtani et al.; (b) Bae et al. (c) Buelga et al.; (d) Bury et al.; (e) Chung et al.; (f) Colin et al.; (g) Garreau et al.; (h) Goti et al.; (i) Huang et al.; (j) Ji et al.; (k) Liu et al.; (l) Medellin-Garibay et al.; (m) Okada et al.; (n) Purwonugroho et al.; (o) Revilla et al.; (p) Shen et al.; (q) Tanaka et al.; (r) Thomson et al.; (s) Tsuda et al.; (t) Vu et al.; (u) Yamamoto et al.; The black solid line and the black dashed line represent the median and the 10th/90th percentile of the dataset, respectively. The grey and light blue area represents the 90% confidence interval of the 50th and 10th/90th quantile of 1000 simulations, respectively.


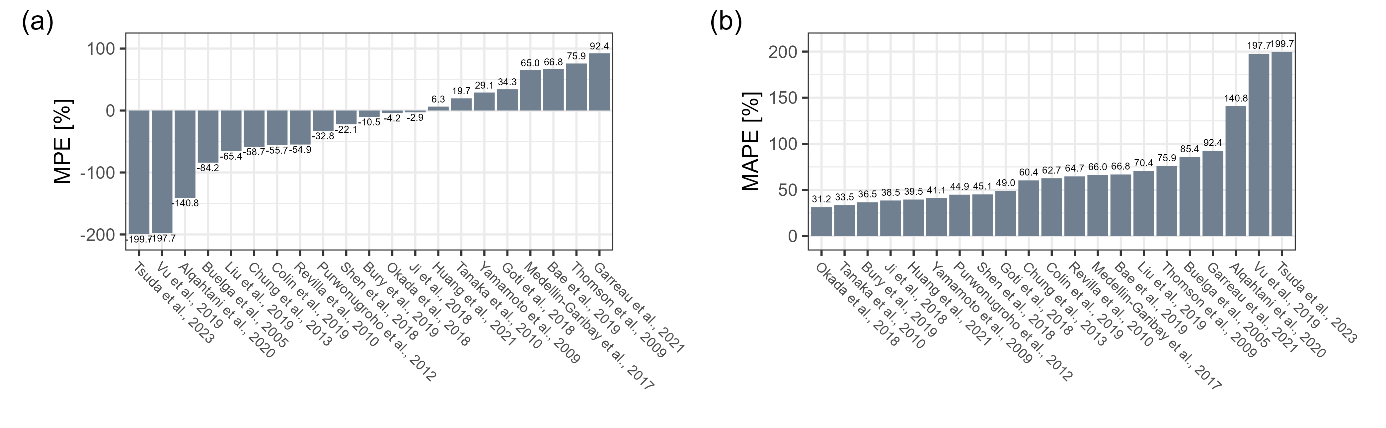


**Figure S2**. (a) Median prediction error (MPE [%]) and (b) median absolute prediction error (MAPE [%]), *a priori* scenario, BMI less than 30.


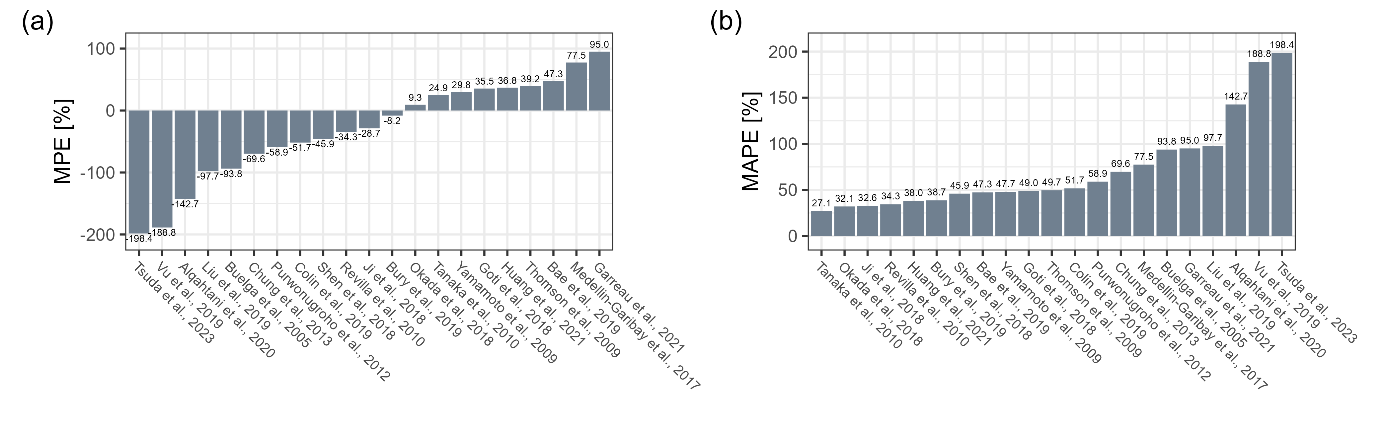


**Figure S3**. (a) Median prediction error (MPE [%]) and (b) median absolute prediction error (MAPE [%]), *a priori* scenario, BMI greater than or equal to 30.


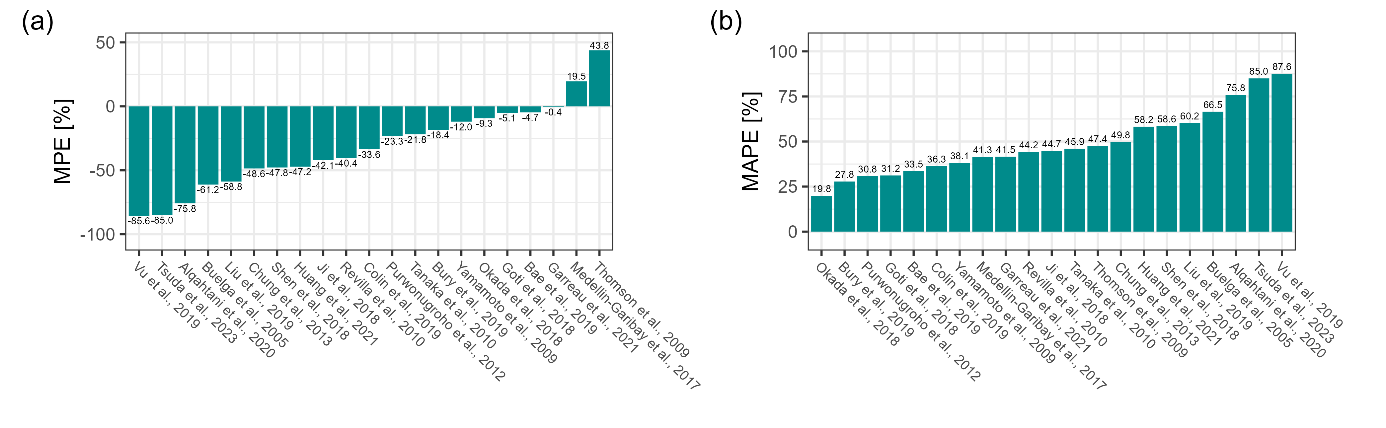


**Figure S4**. (a) Median prediction error (MPE [%]) and (b) median absolute prediction error (MAPE [%]), Bayesian scenario, first prior occasion, BMI less than 30.


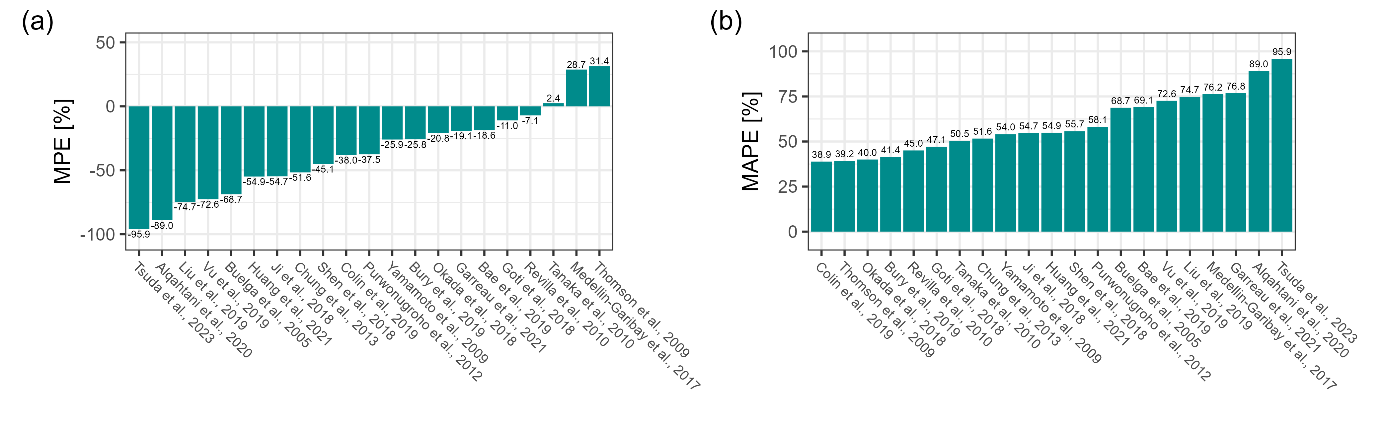


**Figure S5**. (a) Median prediction error (MPE [%]) and (b) median absolute prediction error (MAPE [%]), Bayesian scenario, first prior occasion, BMI greater than or equal to 30.


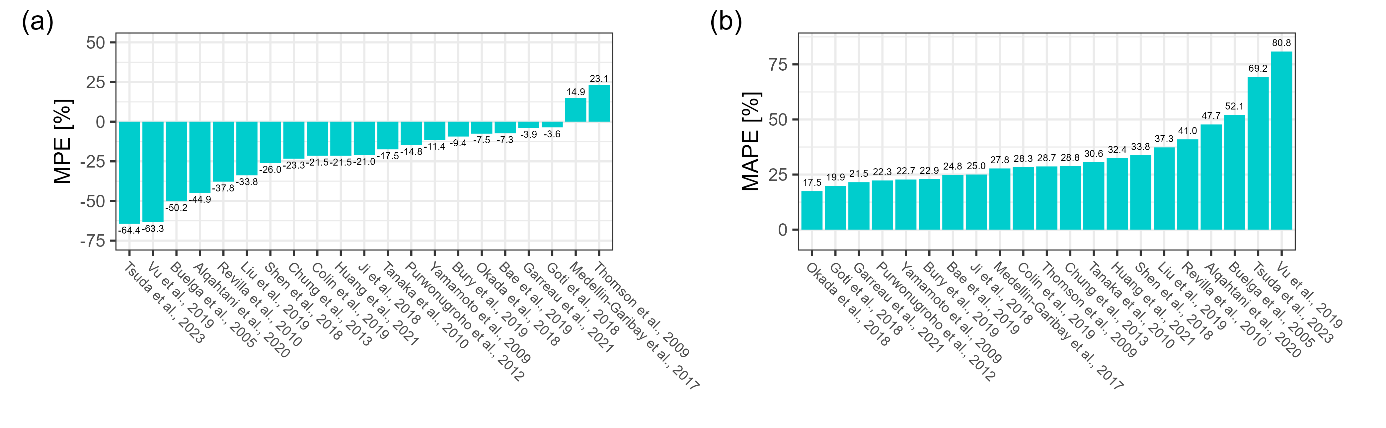


**Figure S6**. (a) Median prediction error (MPE [%]) and (b) median absolute prediction error (MAPE [%]), Bayesian scenario, second prior occasion, BMI less than 30.


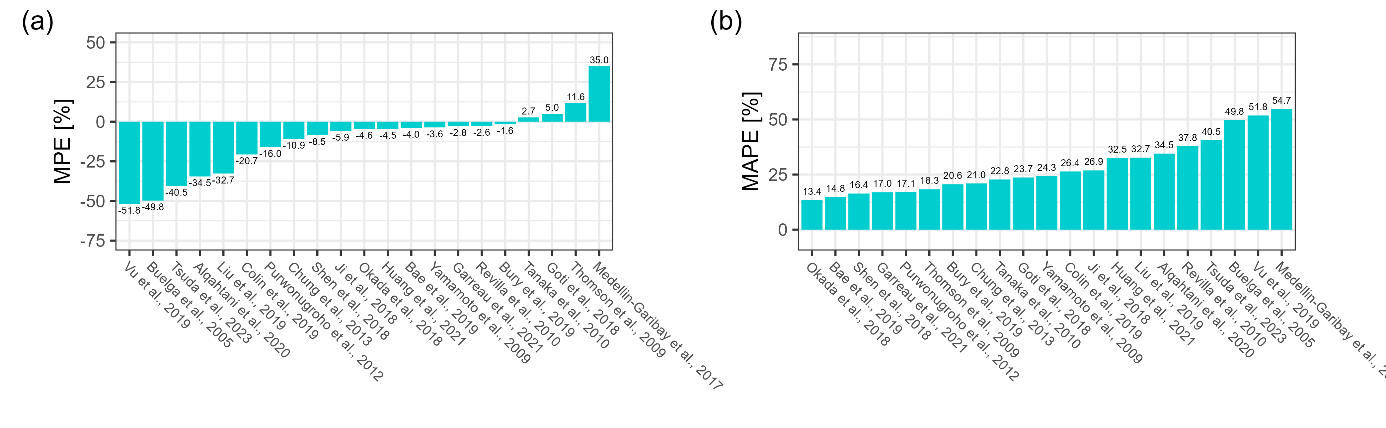


**Figure S7**. (a) Median prediction error (MPE [%]) and (b) median absolute prediction error (MAPE [%]), Bayesian scenario, second prior occasion, BMI greater than or equal to 30.


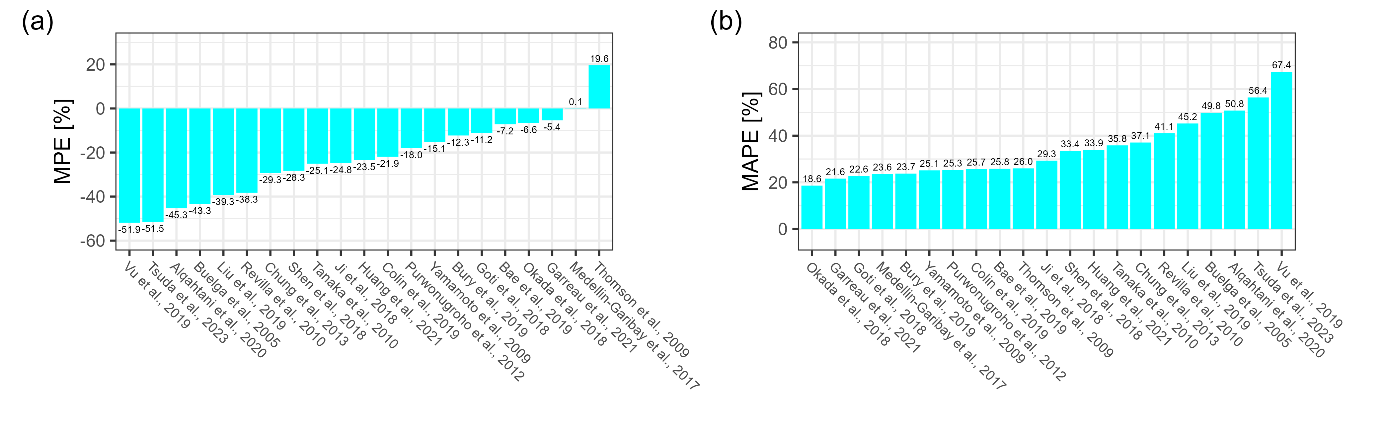


**Figure S8**. (a) Median prediction error (MPE [%]) and (b) median absolute prediction error (MAPE [%]), Bayesian scenario, two prior occasions, BMI less than 30.


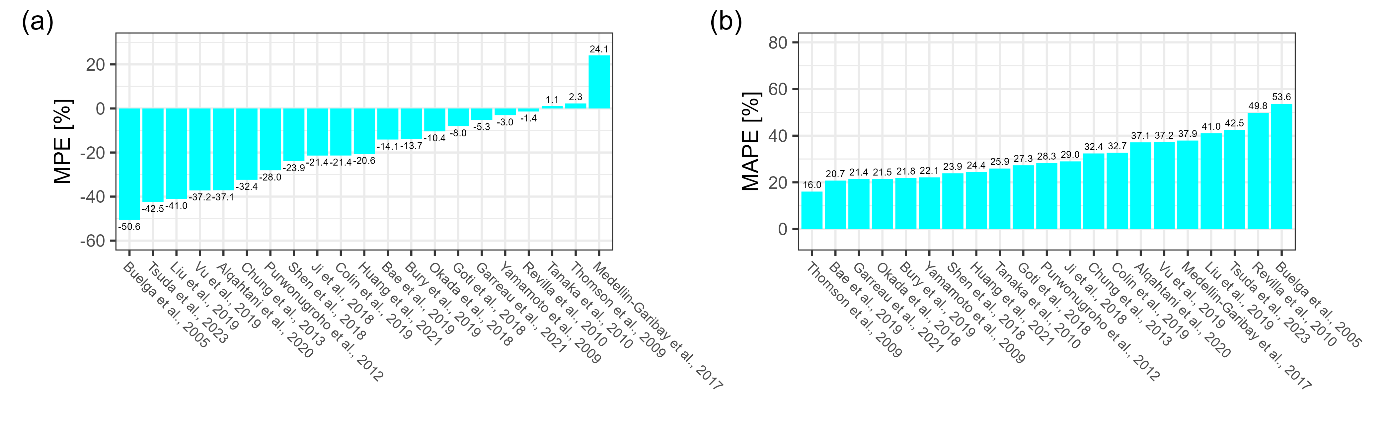


**Figure S9**. (a) Median prediction error (MPE [%]) and (b) median absolute prediction error (MAPE [%]), Bayesian scenario, two prior occasions, BMI greater than or equal to 30.


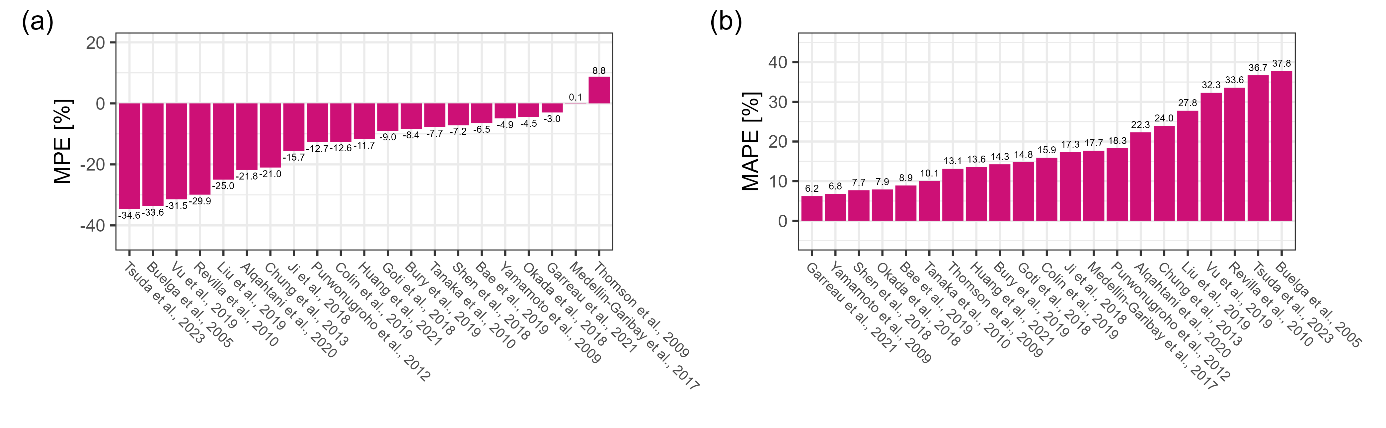


**Figure S10**. (a) Median prediction error (MPE [%]) and (b) median absolute prediction error (MAPE [%]), general model fit scenario, BMI less than 30.


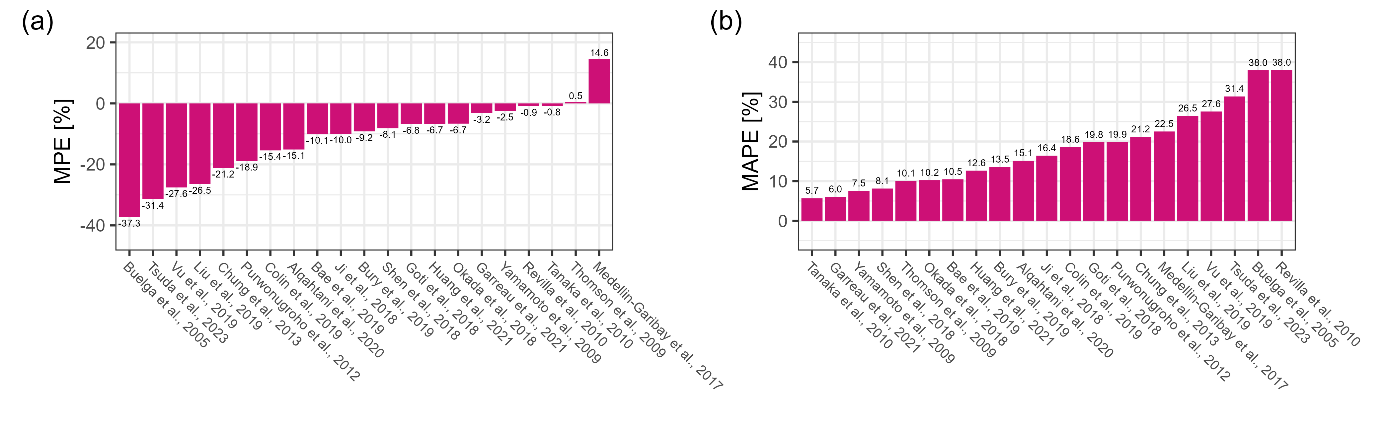


**Figure S11**. (a) Median prediction error (MPE [%]) and (b) median absolute prediction error (MAPE [%]), general model fit scenario, BMI greater than or equal to 30.

References

1. **Abraham J, Sinnollareddy MG, Roberts MS, Williams P, Peake SL, Lipman J, Roberts JA.** 2019. Plasma and interstitial fluid population pharmacokinetics of vancomycin in critically ill patients with sepsis. *Int J Antimicrob Agents* **53:**137-142.

2. **Adane ED, Herald M, Koura F.** 2015. Pharmacokinetics of vancomycin in extremely obese patients with suspected or confirmed Staphylococcus aureus infections. *Pharmacotherapy* **35:**127-139.

3. **Alqahtani SA, Alsultan AS, Alqattan HM, Eldemerdash A, Albacker TB.** 2018. Population Pharmacokinetic Model for Vancomycin Used in Open Heart Surgery: Model-Based Evaluation of Standard Dosing Regimens. *Antimicrob Agents Chemother* **62**.

4. **Bang JY, Kang HI, Lee HJ, Chong YP, Hong SK, Lee EK, Choi BM, Noh GJ.** 2022. Development of a new pharmacokinetic model for target-concentration controlled infusion of vancomycin in critically ill patients. *Clin Exp Pharmacol Physiol* **49:**202-211.

5. **Belabbas T, Yamada T, Egashira N, Hirota T, Suetsugu K, Mori Y, Kato K, Akashi K, Ieiri I.** 2023. Population pharmacokinetic model and dosing optimization of vancomycin in hematologic malignancies with neutropenia and augmented renal clearance. *J Infect Chemother* **29:**391-400.

6. **Belavagi D, Bhandari RK, Shafiq N, Gota V, Patil A, Pandey AK, Mothsara C, Gupta R, Sahni N, Sharma N, Ray P, Kumar V, Sharma SK, Malhotra S.** 2022. A study to explore the appropriateness of dosing regimen of vancomycin in critically ill patients in a tertiary care unit of India. *Germs* **12:**238-252.

7. **Beumier M, Roberts JA, Kabtouri H, Hites M, Cotton F, Wolff F, Lipman J, Jacobs F, Vincent JL, Taccone FS.** 2013. A new regimen for continuous infusion of vancomycin during continuous renal replacement therapy. *J Antimicrob Chemother* **68:**2859-2865.

8. **Bourguignon L, Cazaubon Y, Debeurme G, Loue C, Ducher M, Goutelle S.** 2016. Pharmacokinetics of Vancomycin in Elderly Patients Aged over 80 Years. *Antimicrob Agents Chemother* **60:**4563-4567.

9. **Bue M, Hanberg P, Koch J, Jensen LK, Lundorff M, Aalbaek B, Jensen HE, Soballe K, Tottrup M.** 2018. Single-dose bone pharmacokinetics of vancomycin in a porcine implant-associated osteomyelitis model. *J Orthop Res* **36:**1093-1098.

10. **Chen Z, Taubert M, Chen C, Dokos C, Fuhr U, Weig T, Zoller M, Heck S, Dimitriadis K, Terpolilli N, Kinast C, Scharf C, Lier C, Dorn C, Liebchen U.** 2023. Plasma and Cerebrospinal Fluid Population Pharmacokinetics of Vancomycin in Patients with External Ventricular Drain. *Antimicrob Agents Chemother* **67:**e0024123.

11. **Cheng V, Abdul-Aziz MH, Burrows F, Buscher H, Cho YJ, Corley A, Diehl A, Gilder E, Jakob SM, Kim HS, Levkovich BJ, Lim SY, McGuinness S, Parke R, Pellegrino V, Que YA, Reynolds C, Rudham S, Wallis SC, Welch SA, Zacharias D, Fraser JF, Shekar K, Roberts JA, Investigators AE.** 2022. Population Pharmacokinetics of Vancomycin in Critically Ill Adult Patients Receiving Extracorporeal Membrane Oxygenation (an ASAP ECMO Study). *Antimicrob Agents Chemother* **66:**e0137721.

12. **Coulibaly B, Maire P, Guitton J, Pelletier S, Tangara M, Aulagner G, Goutelle S.** 2023. Population Pharmacokinetics of Vancomycin in Patients Receiving Hemodialysis in a Malian and a French Center and Simulation of the Optimal Loading Dose. *Ther Drug Monit* **45:**637-643.

13. **Deng C, Liu T, Zhou T, Lu H, Cheng D, Zhong X, Lu W.** 2013. Initial dosage regimens of vancomycin for Chinese adult patients based on population pharmacokinetic analysis. *Int J Clin Pharmacol Ther* **51:**407-415.

14. **Dolton M, Xu H, Cheong E, Maitz P, Kennedy P, Gottlieb T, Buono E, McLachlan AJ.** 2010. Vancomycin pharmacokinetics in patients with severe burn injuries. *Burns* **36:**469-476.

15. **Donadello K, Roberts JA, Cristallini S, Beumier M, Shekar K, Jacobs F, Belhaj A, Vincent JL, de Backer D, Taccone FS.** 2014. Vancomycin population pharmacokinetics during extracorporeal membrane oxygenation therapy: a matched cohort study. *Crit Care* **18:**632.

16. **Dorajoo SR, Winata CL, Goh JHF, Ooi ST, Somani J, Yeoh LY, Lee SY, Yap CW, Chan A, Chae JW.** 2019. Optimizing Vancomycin Dosing in Chronic Kidney Disease by Deriving and Implementing a Web-Based Tool Using a Population Pharmacokinetics Analysis. *Front Pharmacol* **10:**641.

17. **Economou CJP, Kielstein JT, Czock D, Xie J, Field J, Richards B, Tallott M, Visser A, Koenig C, Hafer C, Schmidt JJ, Lipman J, Roberts JA.** 2018. Population pharmacokinetics of vancomycin in critically ill patients receiving prolonged intermittent renal replacement therapy. *Int J Antimicrob Agents* **52:**151-157.

18. **Escobar L, Andresen M, Downey P, Gai MN, Regueira T, Borquez T, Lipman J, Roberts JA.** 2014. Population pharmacokinetics and dose simulation of vancomycin in critically ill patients during high-volume haemofiltration. *Int J Antimicrob Agents* **44:**163-167.

19. **Fu X, Lin L, Huang L, Guo L.** 2021. Clinical application of vancomycin population pharmacokinetics model in patients with hematological diseases and neutropenia. *Biopharm Drug Dispos* **42:**427-434.

20. **Glatard A, Bourguignon L, Jelliffe RW, Maire P, Neely MN, Goutelle S.** 2015. Influence of renal function estimation on pharmacokinetic modeling of vancomycin in elderly patients. *Antimicrob Agents Chemother* **59:**2986-2994.

21. **Hamada Y, Kuti JL, Nicolau DP.** 2015. Vancomycin serum concentrations do not adequately predict tissue exposure in diabetic patients with mild to moderate limb infections. *J Antimicrob Chemother* **70:**2064-2067.

22. **Hartinger JM, Michalickova D, Dvorackova E, Hronova K, Krekels EHJ, Szonowska B, Bednarova V, Benakova H, Kroneislova G, Zavora J, Tesar V, Slanar O.** 2023. Intraperitoneally Administered Vancomycin in Patients with Peritoneal Dialysis-Associated Peritonitis: Population Pharmacokinetics and Dosing Implications. *Pharmaceutics* **15**.

23. **He XR, Liu ZH, Ji SM, Liu TT, Li L, Zhou TY, Lu W.** 2014. [Population pharmacokinetics of vancomycin and prediction of pharmacodynamics in the Chinese people]. *Yao Xue Xue Bao* **49:**1528-1535.

24. **He CY, Ye PP, Liu B, Song L, van den Anker J, Zhao W.** 2021. Population Pharmacokinetics and Dosing Optimization of Vancomycin in Infants, Children, and Adolescents with Augmented Renal Clearance. *Antimicrob Agents Chemother* **65:**e0089721.

25. **Heffernan AJ, Germano A, Sime FB, Roberts JA, Kimura E.** 2019. Vancomycin population pharmacokinetics for adult patients with sepsis or septic shock: are current dosing regimens sufficient? *Eur J Clin Pharmacol* **75:**1219-1226.

26. **Hui K, Patel K, Nalder M, Nelson C, Buising K, Pedagogos E, Kong DCM, Kirkpatrick CMJ.** 2019. Optimizing vancomycin dosage regimens in relation to high-flux haemodialysis. *J Antimicrob Chemother* **74:**130-134.

27. **Jaisue S, Pongsakul C, D'Argenio DZ, Sermsappasuk P.** 2020. Population Pharmacokinetic Modeling of Vancomycin in Thai Patients With Heterogeneous and Unstable Renal Function. *Ther Drug Monit* **42:**856-865.

28. **Jing L, Liu TT, Guo Q, Chen M, Lu JJ, Lv CL.** 2020. Development and comparison of population pharmacokinetic models of vancomycin in neurosurgical patients based on two different renal function markers. *J Clin Pharm Ther* **45:**88-96.

29. **Jung Y, Lee DH, Kim HS.** 2021. Prospective Cohort Study of Population Pharmacokinetics and Pharmacodynamic Target Attainment of Vancomycin in Adults on Extracorporeal Membrane Oxygenation. *Antimicrob Agents Chemother* **65**.

30. **Kanji S, Roberts JA, Xie J, Zelenitsky S, Hiremath S, Zhang G, Watpool I, Porteous R, Patel R.** 2020. Vancomycin Population Pharmacokinetics in Critically Ill Adults During Sustained Low-Efficiency Dialysis. *Clin Pharmacokinet* **59:**327-334.

31. **Kim AJ, Lee JY, Choi SA, Shin WG.** 2016. Comparison of the pharmacokinetics of vancomycin in neurosurgical and non-neurosurgical patients. *Int J Antimicrob Agents* **48:**381-387.

32. **Kim DJ, Lee DH, Ahn S, Jung J, Kiem S, Kim SW, Shin JG.** 2019. A new population pharmacokinetic model for vancomycin in patients with variable renal function: Therapeutic drug monitoring based on extended covariate model using CKD-EPI estimation. *J Clin Pharm Ther* **44:**750-759.

33. **Kirwan M, Munshi R, O'Keeffe H, Judge C, Coyle M, Deasy E, Kelly YP, Lavin PJ, Donnelly M, D'Arcy DM.** 2021. Exploring population pharmacokinetic models in patients treated with vancomycin during continuous venovenous haemodiafiltration (CVVHDF). *Crit Care* **25:**443.

34. **Kovacevic T, Miljkovic B, Kovacevic P, Dragic S, Momcicevic D, Avram S, Jovanovic M, Vucicevic K.** 2020. Population pharmacokinetic model of Vancomycin based on therapeutic drug monitoring data in critically ill septic patients. *J Crit Care* **55:**116-121.

35. **Li X, Sun S, Ling X, Chen K, Wang Q, Zhao Z.** 2017. Plasma and cerebrospinal fluid population pharmacokinetics of vancomycin in postoperative neurosurgical patients after combined intravenous and intraventricular administration. *Eur J Clin Pharmacol* **73:**1599-1607.

36. **Li X, Wu Y, Sun S, Zhao Z, Wang Q.** 2016. Population Pharmacokinetics of Vancomycin in Postoperative Neurosurgical Patients and the Application in Dosing Recommendation. *J Pharm Sci* **105:**3425-3431.

37. **Li X, Wu Y, Sun S, Mei S, Wang J, Wang Q, Zhao Z.** 2015. Population Pharmacokinetics of Vancomycin in Postoperative Neurosurgical Patients. *J Pharm Sci* **104:**3960-3967.

38. **Lin WW, Wu W, Jiao Z, Lin RF, Jiang CZ, Huang PF, Liu YW, Wang CL.** 2016. Population pharmacokinetics of vancomycin in adult Chinese patients with post-craniotomy meningitis and its application in individualised dosage regimens. *Eur J Clin Pharmacol* **72:**29-37.

39. **Lin Z, Chen DY, Zhu YW, Jiang ZL, Cui K, Zhang S, Chen LH.** 2021. Population pharmacokinetic modeling and clinical application of vancomycin in Chinese patients hospitalized in intensive care units. *Sci Rep* **11:**2670.

40. **Llopis-Salvia P, Jimenez-Torres NV.** 2006. Population pharmacokinetic parameters of vancomycin in critically ill patients. *J Clin Pharm Ther* **31:**447-454.

41. **Ma KF, Liu YX, Jiao Z, Lv JH, Yang P, Wu JY, Yang S.** 2020. Population Pharmacokinetics of Vancomycin in Kidney Transplant Recipients: Model Building and Parameter Optimization. *Front Pharmacol* **11:**563967.

42. **Mangin O, Urien S, Mainardi JL, Fagon JY, Faisy C.** 2014. Vancomycin pharmacokinetic and pharmacodynamic models for critically ill patients with post-sternotomy mediastinitis. *Clin Pharmacokinet* **53:**849-861.

43. **Medellin-Garibay SE, Ortiz-Martin B, Rueda-Naharro A, Garcia B, Romano-Moreno S, Barcia E.** 2016. Pharmacokinetics of vancomycin and dosing recommendations for trauma patients. *J Antimicrob Chemother* **71:**471-479.

44. **Montanes Pauls B, Alminana MA, Casabo Alos VG.** 2011. Vancomycin pharmacokinetics during continuous ambulatory peritoneal dialysis in patients with peritonitis. *Eur J Pharm Sci* **43:**212-216.

45. **Moore JN, Healy JR, Thoma BN, Peahota MM, Ahamadi M, Schmidt L, Cavarocchi NC, Kraft WK.** 2016. A Population Pharmacokinetic Model for Vancomycin in Adult Patients Receiving Extracorporeal Membrane Oxygenation Therapy. *CPT Pharmacometrics Syst Pharmacol* **5:**495-502.

46. **Mulla H, Pooboni S.** 2005. Population pharmacokinetics of vancomycin in patients receiving extracorporeal membrane oxygenation. *Br J Clin Pharmacol* **60:**265-275.

47. **Munir MM, Rasheed H, Khokhar MI, Khan RR, Saeed HA, Abbas M, Ali M, Bilal R, Nawaz HA, Khan AM, Qamar S, Anjum SM, Usman M.** 2021. Dose Tailoring of Vancomycin Through Population Pharmacokinetic Modeling Among Surgical Patients in Pakistan. *Front Pharmacol* **12:**721819.

48. **Oda K, Jono H, Kamohara H, Nishi K, Tanoue N, Saito H.** 2020. Development of Vancomycin Dose Individualization Strategy by Bayesian Prediction in Patients Receiving Continuous Renal Replacement Therapy. *Pharm Res* **37:**108.

49. **Oda K, Jono H, Saito H.** 2023. Model-Informed Precision Dosing of Vancomycin in Adult Patients Undergoing Hemodialysis. *Antimicrob Agents Chemother* **67:**e0008923.

50. **Parra Gonzalez D, Perez Mesa JA, Cuervo Maldonado SI, Diaz Rojas JA, Cortes JA, Silva Gomez E, Saavedra Trujillo CH, Gomez J.** 2022. Pharmacokinetics of Vancomycin among Patients with Chemotherapy-Associated Febrile Neutropenia: Which Would Be the Best Dosing to Obtain Appropriate Exposure? *Antibiotics (Basel)* **11**.

51. **Radke C, Horn D, Lanckohr C, Ellger B, Meyer M, Eissing T, Hempel G.** 2017. Development of a Physiologically Based Pharmacokinetic Modelling Approach to Predict the Pharmacokinetics of Vancomycin in Critically Ill Septic Patients. *Clin Pharmacokinet* **56:**759-779.

52. **Rao Z, Guo SM, Wei YM.** 2024. Individualized Delivery of Vancomycin by Model-Informed Bayesian Dosing Approach to Maintain an AUC24 Target in Critically Ill Patients. *Chemotherapy* **69:**49-55.

53. **Roberts JA, Taccone FS, Udy AA, Vincent JL, Jacobs F, Lipman J.** 2011. Vancomycin dosing in critically ill patients: robust methods for improved continuous-infusion regimens. *Antimicrob Agents Chemother* **55:**2704-2709.

54. **Sanchez JL, Dominguez AR, Lane JR, Anderson PO, Capparelli EV, Cornejo-Bravo JM.** 2010. Population pharmacokinetics of vancomycin in adult and geriatric patients: comparison of eleven approaches. *Int J Clin Pharmacol Ther* **48:**525-533.

55. **Sansot C, Kalbacher E, Lemoine S, Bourguignon L, Fauvel JP, Ducher M.** 2015. A Bayesian Model to Describe Factors Influencing Trough Levels of Vancomycin in Hemodialysis Patients. *Nephron* **131:**131-137.

56. **Schaedeli F, Uehlinger DE.** 1998. Urea kinetics and dialysis treatment time predict vancomycin elimination during high-flux hemodialysis. *Clin Pharmacol Ther* **63:**26-38.

57. **Sitaruno S, Santimaleeworagun W, Pattharachayakul S, DeBacker KC, Vattanavanit V, Binyala W, Pai MP.** 2022. Comparison of Race-Based and Non-Race-Based Equations for Kidney Function Estimation in Critically Ill Thai Patients for Vancomycin Dosing. *J Clin Pharmacol* **62:**1215-1226.

58. **Smit C, Wasmann RE, Goulooze SC, Wiezer MJ, van Dongen EPA, Mouton JW, Bruggemann RJM, Knibbe CAJ.** 2020. Population pharmacokinetics of vancomycin in obesity: Finding the optimal dose for (morbidly) obese individuals. *Br J Clin Pharmacol* **86:**303-317.

59. **Tsai D, Stewart PC, Hewagama S, Krishnaswamy S, Wallis SC, Lipman J, Roberts JA.** 2018. Optimised dosing of vancomycin in critically ill Indigenous Australian patients with severe sepsis. *Anaesth Intensive Care* **46:**374-380.

60. **Udy AA, Covajes C, Taccone FS, Jacobs F, Vincent JL, Lipman J, Roberts JA.** 2013. Can population pharmacokinetic modelling guide vancomycin dosing during continuous renal replacement therapy in critically ill patients? *Int J Antimicrob Agents* **41:**564-568.

61. **Vazquez M, Fagiolino P, Boronat A, Buroni M, Maldonado C.** 2008. Therapeutic drug monitoring of vancomycin in severe sepsis and septic shock. *Int J Clin Pharmacol Ther* **46:**140-145.

62. **Wang C, Chen J, Yang B, Li S, Zhang Y, Chen L, Wang T, Dong Y.** 2023. Determination of vancomycin exposure target and individualized dosing recommendations for critically ill patients undergoing continuous renal replacement therapy. *Pharmacotherapy* **43:**180-188.

63. **Wang C, Zhang C, Li X, Zhao S, He N, Zhai S, Ge Q.** 2021. Dose Optimization of Vancomycin for Critically Ill Patients Undergoing CVVH: A Prospective Population PK/PD Analysis. *Antibiotics (Basel)* **10**.

64. **Wei S, Zhang D, Zhao Z, Mei S.** 2022. Population pharmacokinetic model of vancomycin in postoperative neurosurgical patients. *Front Pharmacol* **13:**1005791.

65. **Westra N, Proost JH, Franssen CFM, Wilms EB, van Buren M, Touw DJ.** 2019. Vancomycin pharmacokinetic model development in patients on intermittent online hemodiafiltration. *PLoS One* **14:**e0216801.

66. **Wu CC, Shen LJ, Hsu LF, Ko WJ, Wu FL.** 2016. Pharmacokinetics of vancomycin in adults receiving extracorporeal membrane oxygenation. *J Formos Med Assoc* **115:**560-570.

67. **Xu J, Zhu Y, Niu P, Liu Y, Li D, Jiang L, Shi D.** 2022. Establishment and application of population pharmacokinetics model of vancomycin in infants with meningitis. *Pediatr Neonatol* **63:**57-65.

68. **Yellepeddi VK, Lindley B, Radetich E, Kumar S, Bhakta Z, Leclair L, Parrot M, Young DC.** 2024. Population pharmacokinetics and target attainment analysis of vancomycin after intermittent dosing in adults with cystic fibrosis. *Antimicrob Agents Chemother* **68:**e0099223.

69. **Yu Z, Liu J, Yu H, Zhou L, Zhu J, Liang G, Yang Y, Zheng Y, Han Y, Xu J, Han G, Yu L, Zhao Y.** 2023. Population pharmacokinetics and individualized dosing of vancomycin for critically ill patients receiving continuous renal replacement therapy: the role of residual diuresis. *Front Pharmacol* **14:**1298397.

70. **Zaric RZ, Milovanovic J, Rosic N, Milovanovic D, Zecevic DR, Folic M, Jankovic S.** 2018. Pharmacokinetics of Vancomycin in Patients with Different Renal Function Levels. *Open Med (Wars)* **13:**512-519.

71. **Zhao S, He N, Zhang Y, Wang C, Zhai S, Zhang C.** 2021. Population Pharmacokinetic Modeling and Dose Optimization of Vancomycin in Chinese Patients with Augmented Renal Clearance. *Antibiotics (Basel)* **10**.

72. **Zhou Y, Gao F, Chen C, Ma L, Yang T, Liu X, Liu Y, Wang X, Zhao X, Que C, Li S, Lv J, Cui Y, Yang L.** 2019. Development of a Population Pharmacokinetic Model of Vancomycin and its Application in Chinese Geriatric Patients with Pulmonary Infections. *Eur J Drug Metab Pharmacokinet* **44:**361-370.

73. **Alqahtani S, Almatrafi A, Bin Aydan N, Alqahtani M, Alzamil F, Alsultan A, Asiri Y.** 2020. Optimization of Vancomycin Dosing Regimen in Cancer Patients using Pharmacokinetic/Pharmacodynamic Modeling. *Pharmacotherapy* **40:**1192-1200.

74. **Bae SH, Yim DS, Lee H, Park AR, Kwon JE, Sumiko H, Han S.** 2019. Application of Pharmacometrics in Pharmacotherapy: Open-Source Software for Vancomycin Therapeutic Drug Management. *Pharmaceutics* **11**.

75. **Buelga DS, del Mar Fernandez de Gatta M, Herrera EV, Dominguez-Gil A, Garcia MJ.** 2005. Population pharmacokinetic analysis of vancomycin in patients with hematological malignancies. *Antimicrob Agents Chemother* **49:**4934-4941.

76. **Bury D, Ter Heine R, van de Garde EMW, Nijziel MR, Grouls RJ, Deenen MJ.** 2019. The effect of neutropenia on the clinical pharmacokinetics of vancomycin in adults. *Eur J Clin Pharmacol* **75:**921-928.

77. **Chung JY, Jin SJ, Yoon JH, Song YG.** 2013. Serum cystatin C is a major predictor of vancomycin clearance in a population pharmacokinetic analysis of patients with normal serum creatinine concentrations. *J Korean Med Sci* **28:**48-54.

78. **Colin PJ, Allegaert K, Thomson AH, Touw DJ, Dolton M, de Hoog M, Roberts JA, Adane ED, Yamamoto M, Santos-Buelga D, Martin-Suarez A, Simon N, Taccone FS, Lo YL, Barcia E, Struys M, Eleveld DJ.** 2019. Vancomycin Pharmacokinetics Throughout Life: Results from a Pooled Population Analysis and Evaluation of Current Dosing Recommendations. *Clin Pharmacokinet* **58:**767-780.

79. **Garreau R, Falquet B, Mioux L, Bourguignon L, Ferry T, Tod M, Wallet F, Friggeri A, Richard JC, Goutelle S.** 2021. Population Pharmacokinetics and Dosing Simulation of Vancomycin Administered by Continuous Injection in Critically Ill Patient. *Antibiotics (Basel)* **10**.

80. **Goti V, Chaturvedula A, Fossler MJ, Mok S, Jacob JT.** 2018. Hospitalized Patients With and Without Hemodialysis Have Markedly Different Vancomycin Pharmacokinetics: A Population Pharmacokinetic Model-Based Analysis. *Ther Drug Monit* **40:**212-221.

81. **Huang J, Wang X, Hao C, Yang W, Zhang W, Liu J, Qu H.** 2021. Cystatin C and/or creatinine-based estimated glomerular filtration rate for prediction of vancomycin clearance in long-stay critically ill patients with persistent inflammation, immunosuppression and catabolism syndrome (PICS): a population pharmacokinetics analysis. *Intern Emerg Med* **16:**1883-1893.

82. **Ji XW, Ji SM, He XR, Zhu X, Chen R, Lu W.** 2018. Influences of renal function descriptors on population pharmacokinetic modeling of vancomycin in Chinese adult patients. *Acta Pharmacol Sin* **39:**286-293.

83. **Liu TT, Pang HM, Jing L, Wei WX, Qin XL, Guo Q, Lu H, Cheng DH, Jiang WZ.** 2019. A population pharmacokinetic model of vancomycin for dose individualization based on serum cystatin C as a marker of renal function. *J Pharm Pharmacol* **71:**945-955.

84. **Medellin-Garibay SE, Romano-Moreno S, Tejedor-Prado P, Rubio-Alvaro N, Rueda-Naharro A, Blasco-Navalpotro MA, Garcia B, Barcia E.** 2017. Influence of Mechanical Ventilation on the Pharmacokinetics of Vancomycin Administered by Continuous Infusion in Critically Ill Patients. *Antimicrob Agents Chemother* **61**.

85. **Okada A, Kariya M, Irie K, Okada Y, Hiramoto N, Hashimoto H, Kajioka R, Maruyama C, Kasai H, Hamori M, Nishimura A, Shibata N, Fukushima K, Sugioka N.** 2018. Population Pharmacokinetics of Vancomycin in Patients Undergoing Allogeneic Hematopoietic Stem-Cell Transplantation. *J Clin Pharmacol* **58:**1140-1149.

86. **Purwonugroho TA, Chulavatnatol S, Preechagoon Y, Chindavijak B, Malathum K, Bunuparadah P.** 2012. Population pharmacokinetics of vancomycin in Thai patients. *ScientificWorldJournal* **2012:**762649.

87. **Revilla N, Martin-Suarez A, Perez MP, Gonzalez FM, Fernandez de Gatta Mdel M.** 2010. Vancomycin dosing assessment in intensive care unit patients based on a population pharmacokinetic/pharmacodynamic simulation. *Br J Clin Pharmacol* **70:**201-212.

88. **Shen K, Yang M, Fan Y, Liang X, Chen Y, Wu J, Yu J, Zhang H, Wang R, Zhang F, Hang J, Wen X, Li H, Shen L, Zhang Z, Wu S, Shen B, Huang W, Chang C, Shen Y, Ren H, Yuan Q, Song X, Luo X, Zhang H, Yang W, Yang J, Zhang J.** 2018. Model-based Evaluation of the Clinical and Microbiological Efficacy of Vancomycin: A Prospective Study of Chinese Adult In-house Patients. *Clin Infect Dis* **67:**S256-S262.

89. **Tanaka A, Aiba T, Otsuka T, Suemaru K, Nishimiya T, Inoue T, Murase M, Kurosaki Y, Araki H.** 2010. Population pharmacokinetic analysis of vancomycin using serum cystatin C as a marker of renal function. *Antimicrob Agents Chemother* **54:**778-782.

90. **Thomson AH, Staatz CE, Tobin CM, Gall M, Lovering AM.** 2009. Development and evaluation of vancomycin dosage guidelines designed to achieve new target concentrations. *J Antimicrob Chemother* **63:**1050-1057.

91. **Tsuda Y, Takahashi M, Watanabe F, Goto K, Echizen H.** 2023. Population Pharmacokinetic Analysis of Vancomycin in Patients with Solid or Hematological Malignancy in Relation to the Quick Sequential Organ Failure Assessment Scores. *Eur J Drug Metab Pharmacokinet* **48:**647-655.

92. **Vu DH, Nguyen DA, Delattre IK, Ho TT, Do HG, Pham HN, Dao XC, Tran NT, Nguyen GB, Van Bambeke F, Tulkens PM, Nguyen HA.** 2019. Determination of optimal loading and maintenance doses for continuous infusion of vancomycin in critically ill patients: Population pharmacokinetic modelling and simulations for improved dosing schemes. *Int J Antimicrob Agents* **54:**702-708.

93. **Yamamoto M, Kuzuya T, Baba H, Yamada K, Nabeshima T.** 2009. Population pharmacokinetic analysis of vancomycin in patients with gram-positive infections and the influence of infectious disease type. *J Clin Pharm Ther* **34:**473-483.
